# Evaluation of the relative efficacy of a couple cognitive-behaviour therapy (CBT) for Premenstrual Disorders (PMDs), in comparison to one-to-one CBT and a wait list control: A randomized controlled trial

**DOI:** 10.1371/journal.pone.0175068

**Published:** 2017-04-18

**Authors:** Jane M. Ussher, Janette Perz

**Affiliations:** Translational Health Research Institute (THRI), School of Medicine, Western Sydney University, Sydney, New South Wales, Australia; University of Marburg, GERMANY

## Abstract

**Design:**

A randomised control trial (RCT) was conducted to examine the efficacy of couple-based cognitive behaviour therapy (CBT) for Premenstrual Disorders (PMDs), in comparison to one-to-one CBT and a wait-list control.

**Methods:**

Triangulation of quantitative and qualitative outcome measures evaluated changes pre-post intervention. Eighty three women were randomly allocated across three conditions, with 63 completing post-intervention measures, a retention rate of 76%.

**Results:**

Repeated measures analysis of variance found a significant time by group interaction identifying that women in the two CBT conditions reported lower total premenstrual symptoms, emotional reactivity/mood, and premenstrual distress, in comparison to the wait list control. Significantly higher active behavioural coping post-intervention was found in the couple condition than in the one-to-one and wait list control groups. Qualitative analysis provided insight into the subjective experience of PMDs and participation in the intervention study. Across groups, women reported increased awareness and understanding of premenstrual change post-intervention. A larger proportion of women in the CBT conditions reported reduction in intensity and frequency of negative premenstrual emotional reactivity, increased communication and help-seeking, increased understanding and acceptance of embodied change, and the development of coping skills, post-intervention. Increased partner understanding and improved relationship post-intervention was reported by a greater proportion of participants in the CBT conditions, most markedly in the couple condition.

**Conclusion:**

These findings suggest that one-to-one and couple CBT interventions can significantly reduce women’s premenstrual symptomatology and distress, and improve premenstrual coping. Couple based CBT interventions may have a greater positive impact upon behavioural coping and perceptions of relationship context and support. This suggests that CBT should be available for women reporting moderate-severe PMDs, with couple-based CBT offering additional benefits to a one-to-one modality.

## Introduction

Premenstrual change is experienced by up to 90% of women, with up to 40% experiencing moderate distress, categorised by clinicians and researchers as Premenstrual Syndrome (PMS) [1], and 2–5% experiencing severe distress and disruption to their lives, categorised as Premenstrual Dysphoric Disorder (PMDD) [2]. Recognition of the continuum of premenstrual distress, and overlap between the diagnostic categories PMS and PMDD, has led to the adoption of the term ‘Premenstrual Disorders’ (PMDs) by an expert advisory panel [3]. PMDs include emotional and behavioural symptoms that have a significant impact on a woman’s quality of life during the premenstrual phase of the cycle, but are absent after menstruation and before ovulation. The symptoms most commonly reported include irritability, depression, mood swings, anxiety, concentration difficulties, feelings of loss of control and tiredness, often combined with physical symptoms such as bloating, breast tenderness, headache and general body aches [4].

The costs of PMDs, in terms of impact upon women’s quality of life and economic functioning, are estimated to be considerable [1, 5]. This has led to the development of a range of biomedical interventions, including the use of anti-depressants of the serotonin reuptake inhibitor (SSRI) class, anxiolytics and hormone treatments to suppress ovulation, and oophorectomy [3, 4]. Although these approaches may be effective in reducing premenstrual symptoms, they do not take account of the complex mechanisms underlying premenstrual distress that are not adequately accounted for by physiology alone [6, 7]. Furthermore, many women express a preference for non-medical treatment options for their premenstrual symptoms [8], due to side effects [9], or contraindications to drug treatments [10]. Consequently, there has been a development of psychological approaches to PMDs treatment [11], that take into account the interaction of embodied, cognitive and socio-cultural factors in the development of symptoms [12].

The results of systematic review [10] and meta-analysis of randomized controlled trials [13, 14] suggest that cognitive behaviour therapy (CBT) can reduce premenstrual anxiety and depression, have a beneficial impact on behavioural change, and reduce interference of premenstrual symptoms on daily living [15, 16–18]. Such interventions involve a combination of behavioural strategies such as relaxation training, coping skills, social support, and anger management [19–21], combined with facilitation of cognitive restructuring to overcome the sense of helplessness associated with premenstrual symptoms and reframe self-defeating cognitions [11, 22]. CBT has been demonstrated to be as effective as SSRI’s in reducing premenstrual distress in the short-term, and at long term follow-up to be more effective, in terms of reducing premenstrual distress and improving coping [14, 17].

### Relational context of premenstrual distress

A limitation of existing psychological or medical interventions for PMDs is that the focus is on the woman who reports distress. This negates research evidence that has found that premenstrual distress is often a relational experience, developing, and being positioned as ‘PMS’, within the context of interactions with partners or children [23]. Using a short fuse metaphor, women report greater reactivity to family stresses and altered perception of daily life stresses premenstrually [24, 25].Women, and their families, may also attribute premenstrual expression of negative emotion to ‘PMS’, even when alternative explanations can be found, which leads to women’s emotions being positioned as a hormonal pathology [12]. Direct expression of emotion has been reported to be lower in families where women report PMDs [26], which increases the likelihood of premenstrual emotion being positioned as problematic.

Many women who report PMDs also report higher levels of relationship dissatisfaction or difficulties [26–31]. There is evidence that both women and their partners evaluate the relationship more negatively in the premenstrual phase, suggesting that some couples are not simply distressed, but rather, are distressed in the luteal phase of the cycle [32, 33]. It has also been reported that premenstrual anger and irritation is associated with legitimate relationship conflicts, with feelings of dissatisfaction being openly expressed during the premenstrual phase of the cycle [34, 35]. This is in contrast to women’s self-silencing during the remainder of the month, where they take up a position of self-sacrifice and self-renunciation [36], in an attempt to live up to idealised notions of femininity [37]. Whilst some women describe the premenstrual expression of negative emotion as cathartic in the short term, this catharsis is invariably followed by guilt, self-criticism, and a positioning of the premenstrual self as ‘out of control’ [38, 39]. It is thus the *break* in self-silencing premenstrually, described as a transgression from the ‘real me’, which leads to distress, as well as to self-castigation [36].

The role of partners in the exacerbation or amelioration of premenstrual distress has been demonstrated in a number of studies. The coping responses of men have been found to be a strong predictor of women’s symptom severity, with high levels of premenstrual distress associated with a partner’s avoidance, fear, and anger, and low levels of distress associated with reassurance and support [7, 40, 41]. Men’s constructions of ‘PMS’ have been implicated in women’s negative premenstrual experiences, with evidence that many men treat women with PMDs in a belittling way [42, 43]. At the same time, some male partners of women with PMDs report that they experience moderate to significant disruption in their lives due to their partner’s premenstrual change [30, 32], or report that they wished they had married someone else [44], describing the premenstrual period as like walking on eggshells [36].

The findings of the above body of research have led to the suggestion that PMDs is not an individual problem, but a relational issue [12, 33], and that coping with moderate-severe PMDs requires effort from both members of a couple [41]. In this vein, family or couple therapy has been suggested as an appropriate form of intervention for PMDs [45]. In couple therapy, it is not necessarily the relationship that becomes the focus of treatment, rather, the involvement of the partner in the therapy process enhances symptom alleviation and reduces relationship distress [46]. Frank and colleagues [30] investigated the impact of including male partners in the monitoring of a woman’s behavioural and emotional premenstrual symptoms and reported significant improvement in relationship functioning and reduction in premenstrual distress compared to a self-monitoring control group. Awareness of cyclical patterns meant that couples could develop joint strategies to deal with potential problems, and discuss major issues at times other than the premenstrual phase. A subsequent small scale couple intervention, comparing a PMDs and non-PMDs control, reported positive effects of time, but no difference between groups [47]. There have been no other systematic evaluations of couple-based interventions for PMDs, and no comparisons of the efficacy of individual and couple CBT interventions in reducing premenstrual distress. Previous research on the efficacy of CBT in reducing premenstrual distress has also been criticised for the absence of wait-list control groups, small sample size, absence of random group assignment, and failure to use using repeated measures ANOVA to assess main effects of group, main effects of time, and time by group interactions [10]. There is also an absence of a qualitative examination of the subjective experience and mechanisms of change pre-post intervention, with the exception of one study [48]. There has been a call for the addition of qualitative methods to RCT’s, in order to address complexity, context, and meaning of change, along-side quantitative measures of outcome [49]

The aim of the present study was to address these gaps in the research literature, through the evaluation of the relative efficacy of a couple-based CBT intervention in comparison to a one-to-one CBT intervention and a wait-list control, in reducing premenstrual symptoms and distress, and in improving premenstrual coping, using a randomised controlled trial, and a triangulation of outcome measures. The impact of the interventions on depression and anxiety, self-silencing, and relationship adjustment were also examined, to ascertain changes in general wellbeing, relationship functioning and communication.

## Materials and methods

### Design

A randomised control trial (RCT) was conducted to examine the relative efficacy of a brief couple-based intervention for PMDs, in comparison to a proven one-to-one CBT PMDs intervention and a wait-list control. This allows for the comparison of active treatment with absence of treatment, and the comparison of two active treatments. Triangulation of quantitative and qualitative outcome measures was used to evaluate changes pre-post intervention.

### Participants and procedure

Women who reported moderate-severe PMDs were recruited from a range of contexts, including social media, sexual and reproductive health clinics, local radio and newspapers, and women’s health centres. Women who were interested in the study were informed about the nature of the study, and then completed an on-line survey which examined demographic variables, the nature and degree of their premenstrual distress, degree of premenstrual coping, and partner support. Participants were eligible if they were aged between 18–45 years, having regular cycles (21–35 days); presently not taking hormonal medication (excluding contraceptives), psychotropic medication, or having been diagnosed with a major psychiatric illness; not having been pregnant or lactating within the previous 12 months. Criteria for a PMDs diagnosis were assessed with the Premenstrual Symptoms Screening Tool (PSST) [50], with confirmation by daily diary measures [51], demonstrating a 30% difference in symptoms between the pre- and post-menstrual period, for two consecutive months, which cause moderate-severe impairment [3, 52, 53]. If women did not report a 30% increase in premenstrual symptoms after two cycles, they were invited to complete a third, and sometimes fourth, cycle of daily diaries. The baseline pre-screening survey was completed by 1124 participants, with 584 participants invited to complete daily diaries (Table 1). Those who met the criteria for a PMDs diagnosis following daily dairies and agreed to take part (N = 96) were then randomly allocated to one of three treatment groups—couple-based, one-to-one, or wait-list control. Minimum sample sizes of 30 women per group have been recommended for PMS research [54] and ‘large samples’ have been suggested for research examining the relative efficacy of couple therapy [46]. An a priori sample calculation analysis on G*Power for a repeated measures ANOVA (incorporating interaction effects) for 3 groups, a power of 0.95, a two-way alpha level of 0.01 to adjust for multiple comparisons, and a medium effect size (*f* = .25), computed a required total sample size of 90. Generation of the random allocation sequence, using permuted block randomization [55], was conducted by the second author (JP). Allocation of participants to conditions was conducted by a researcher who was not involved in the delivery of intervention. Participants were recruited between 1^st^ March 2012 and 31^st^ December 2012, with quantitative and qualitative data collected between 1^st^ March 2012 and 7^th^ May 2014.

**Table 1:** Demographic characteristics of women in the intervention groups.

	Couple		One-to-One		Wait-List Control		Test	Sig.	Effect Size
Variable	*n*	*M(SD)*	*n*	*M(SD)*	*n*	*M(SD)*	*F*	*p*	*η*^*2*^
Patient age	28	35.14(7.67)	30	34.67(8.07)	25	34.56(8.29)	.041	.960	.001
Relationship Length	28	8.30(7.732)	30	9.97(6.81)	25	9.56(7.52)	.402	.670	.010
	*n*	*%*	*n*	*%*	*n*	*%*	*χ*^*2*^	*p*	*ϕ*
Relationship Status									
Partnered/living together	25	89.3	25	83.3	22	88.0	3.120	.538	.194
Partnered/not living together	2	7.1	5	16.7	3	12.0			
Other	1	3.6	-	-	-	-			
Contraceptive use									
None	12	42.9	13	43.3	5	20.0	6.007	.199	.269
Hormonal^a^	6	21.4	3	10.0	4	16.0			
Other^b^	10	35.7	14	46.7	16	64.0			
Sexuality									
Heterosexual	28	100	30	100	25	100			

^a^ Includes oral contraceptive pill, intra-uterine hormonal device or implant

^b^ Includes condoms, abstinence, sterilization or withdrawal.

Randomised participants completed a series of questionnaires and open ended survey items immediately prior to study entry, at post-intervention (5 months), and for both treatment groups, at three-month follow-up. This included measures of premenstrual symptoms, premenstrual distress and coping, depression and anxiety, relationship adjustment, and self-silencing. Ten women from each of the three conditions were interviewed pre- and post-intervention, and ten from each of the treatment groups interviewed at follow-up. Following Blake and colleagues [18], participants in the wait list control condition were not required to complete the three-month follow-up questionnaires or interviews for ethical considerations. All wait list participants were provided with a previously validated self-help CBT package post-intervention [56], and invited to take part in a group meeting with a clinical psychologist to discuss their PMDs. As a result of a procedural oversight, the authors note that the study was not publically registered before participant enrolment had begun. The study has been retrospectively registered with the Australian New Zealand Clinical Trials Registry (ANZCTR), ACTRN: ACTRN12616000932460 and no changes were made to the study protocol or outcomes after the trial commenced. The authors confirm that there are no ongoing or related trials for this intervention. Written informed consent was obtained from all participants, and ethics approval was received from the Western Sydney University Human Research Ethics Committee (31^st^ January 2012, H6698).

### Interventions

The one-to-one intervention was based on a woman-centred PMDs CBT intervention previously demonstrated to be effective in reducing premenstrual distress in both a face-to face [17] and self-help modality [56]. It consisted of four 90-minute sessions, conducted over a five-month period (three sessions offered on a monthly basis, and one session at two month follow-up), delivered by a woman clinical psychologist. The aims of the intervention were to examine women’s attributions for premenstrual distress within a bio-psycho-social framework, and to challenge negative self-blaming beliefs that may exacerbate symptomatology, such as “I should be calm and controlled all of the time”, “I should always be able to cope”, “I shouldn’t be angry or irritable”. Behavioural coping skills, including relaxation training, taking time out for self-care, diet and exercise were also examined and encouraged across the menstrual cycle. Finally, the relational context of premenstrual distress was explored, and assertiveness training techniques used to encourage calm expression of emotion, concerns or needs throughout the month [22]. Women were given homework following each of the sessions and a written booklet containing detailed information about each session, to supplement the meetings with the psychologist. The couple intervention followed the same format, with inclusion of Couples Dialogue techniques [57] to facilitate couple communication. This allows for the active involvement of the woman’s partner in understanding PMDs, and in strategies of prevention and amelioration.

### Measures

**Premenstrual Symptoms Screening Tool (PSST)** [50] is a 19 item measure used to identify women who suffer from severe PMDs and who are likely to benefit from treatment. For this study, premenstrual symptoms were treated as a continuous variable to allow for changes in the degree of severity to be assessed. A principal components analysis was run on the 14 premenstrual symptoms at pre-test for all participants. A three-component solution explained 67.14% of the total variance and varimax orthogonal rotation was used to aid interpretability. The factors were interpreted as the subscales: Emotional reactivity/mood (5 items); Lack of energy/interest (5 items); and Physical symptoms (4 items). Scores for each subscale were calculated by summing raw scores corresponding to all items loading on a factor along with a total symptom score. The identified symptom subscale profiles were similar to those found in previous factor analyses with the PSST [58].

**Subjective Evaluation of PMDs questionnaire (SEPQ)** [56] examines experiences of premenstrual change two 10 point Likert scales: “To what extent do you find your PMS distressing?” and “To what extent do you feel that you can deal with your PMS?”

*The Premenstrual Coping Checklist (PMCC)* [59] is comprised of 38 items which reflect four principle premenstrual coping strategies termed active-behavioural, active-cognitive, avoidance and menstrual cycle specific (scale of 1–4).

**Hospital Anxiety and Depression Scale (HADS)** [60], is a 14 item validated self-report measure developed to measure anxiety (HADSA) and depression (HADSD) in non-psychiatric populations. A score of between 8 and above is recommended for “caseness”, the cut-off for clinical diagnosis [61].

**The Dyadic Adjustment Scale (DAS)** [62] is a 32-item self-report scale that assesses the quality of couple relationships. Results are summated with high scores indicating greater satisfaction and low scores conflict between the couple.

**Silencing the Self Scale (STSS)** [63, 64] is a standardised questionnaire consisting of 31 items measuring the extent to which individuals endorse self-silencing thoughts and actions, using a 5 point Likert scale. In addition to a Global score, the four subscales are: Care as Self-Sacrifice; Silencing the Self; Externalised Self Perception; and The Divided Self.

#### Subjective experience of premenstrual change and the intervention

In the open ended survey items, participants were asked about their perception of premenstrual change at pre and post intervention, and their experiences of participating in the treatment program, in relation to feelings about other people, feelings about the body, relationship issues associated with premenstrual feelings, and post-intervention, positive consequences of the study.

#### Interviews

Interviews are increasingly being used in the social and behavioural sciences as a means of examining the subjective construction and meaning of experience [65, 66]. Semi-structured one-to-one interviews were conducted with 10 women randomly selected from each of the three conditions, pre and post-intervention. A sample size of 10 was deemed sufficient to reach saturation (no new themes in three consecutive interviews), and follows recommendations for sample size for qualitative research [66]. The interview schedule has been previously used to examine moderate-severe PMS in both the UK and Australia [67–69], and asked women to describe the course and development of premenstrual distress and recount a typical experience of ‘PMS’ in the context of relationships. Post-intervention, the same questions were asked, with a focus on how the intervention has impacted in each of these areas. Interviews were digitally recorded and took approximately 60 minutes.

### Analysis

#### Statistical analysis

Univariate analyses were conducted to compare participants in the three intervention conditions for each of the socio-demographic variables of interest. For continuous variables, one-way ANOVA were conducted with intervention used as the grouping variable, and the chi square test for independence used for frequency data. Descriptive frequency analyses were used to examine baseline/follow-up retention rates across intervention conditions. For each intervention condition, one-way analysis of variance (ANOVA) (status: completer vs. non-completer) was conducted with pre-test outcome variables. Chi square analyses were used to assess group differences in PMDs cases at post-intervention, and at follow-up in the two active conditions. One-way ANOVA (intervention: couple-based vs. one-to-one vs. wait-list control) was performed on outcome variables to assess baseline differences between the intervention groups. Due to violations in the assumptions of homogeneity of regression coefficients for the outcome measures, analysis of covariance (ANCOVA) with pre-test scores as covariates was not used. A series of mixed 2 (time: pre vs. post) x 3 (intervention: couple-based vs. one-to-one vs. wait-list control) ANOVA were conducted for each outcome variable. Follow-up comparisons for each outcome variable were tested with separate mixed 2 (time: post vs. follow-up) x 2 (intervention: couple-based vs. one-to-one) ANOVA. Where significant interaction effects were found, the simple main effect of intervention group was tested with one-way ANOVA, and repeated measures ANOVA for time. The expectation–maximization (EM) algorithm was used to estimate missing data in the ANOVA models tested. An alpha level of .01 was used to adjust for multiple comparisons in the primary statistical tests, whereas .05 was used for all other statistical tests. IBM SPSS 24 statistical software was used for the analyses.

#### Qualitative analysis

Thematic analysis [70] was used to analyse the open ended survey responses and interviews. This involved independent reading of responses to each question by two members of the research team, in order to ascertain the major themes emerging, and to develop a coding frame, based on notions of consistency, commonality, and the function and effects of specific themes. The entire data set was then coded, using NVivo–a software package that assists with the organisation and analysis of textual data. Following coding, the percentage of responses for the open ended survey responses was calculated within each theme, across intervention conditions, to ascertain the magnitude of changes pre-post intervention within and between groups. The open ended survey items were double-blind coded; interrater reliability was high (Cohen’s Kappa = .89). Conceptually clustered matrices [71] were used to organise and display the main themes that emerge from the open ended survey items pre-post intervention.

## Results

### Participant profile

**Fig 1:**
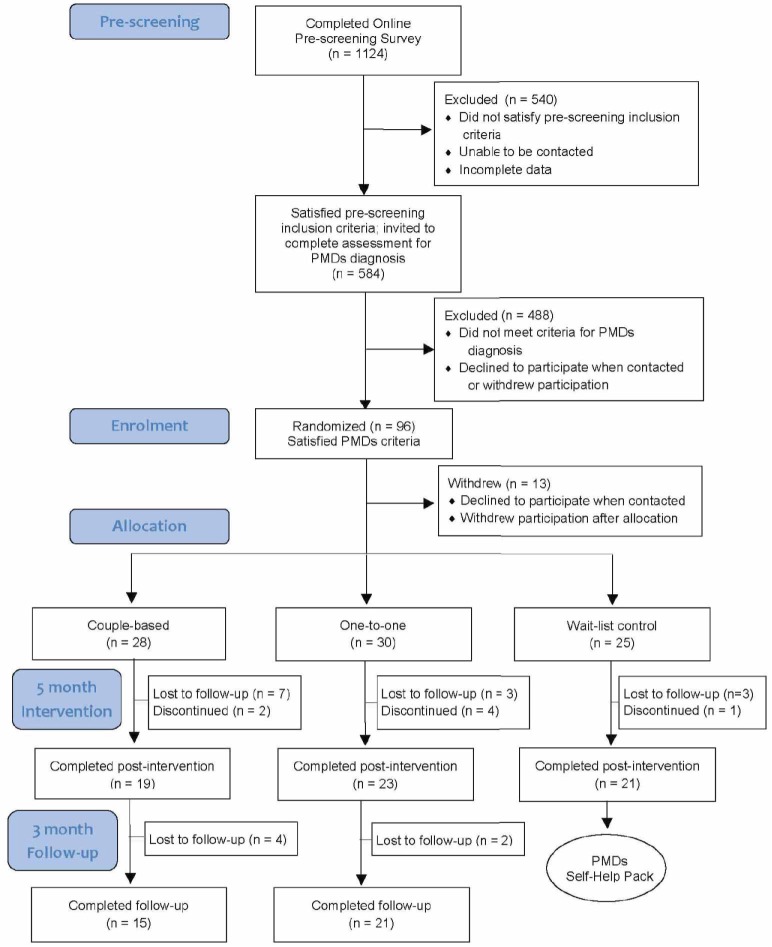
Participant flow diagram.

Ninety six women who satisfied PMDs criteria and agreed to take part in the intervention with their partners were allocated into one of the three intervention conditions (Fig 1). Thirteen women declined to participate following allocation, for reasons including change in circumstances and dissatisfaction with the intervention condition they had been allocated to. Of the 83 participants who commenced the study, 63 completed post measures representing an overall retention rate of 76% post-test. The retention to follow-up was higher in the one-to-one group (70%) compared to 54% for the couple-based group.

Baseline participant demographic data for the 83 women who completed pre-intervention measures is presented in Table 1 by group. The average age of women (mean (*M)* = 34.79, standard deviation (*SD)* = 8.01) did not significantly differ between groups. Eighty two participants were heterosexual, and one identified as lesbian. All women were partnered and with the majority living with their partner, and had an average length of current relationship of 9.27 years (*SD* = 7.32). Contraceptive use was varied across the sample, but did not significantly differ between groups. No statistical differences on pre-test outcome variables were found between participants who completed the interventions and assessments and non-completers (lost to follow-up and discontinued) in each intervention group

### Group baseline comparisons

One-way ANOVA for pre-test measures showed no significant differences between participants in the three intervention groups (*p* values ≥ .17) as reported in Table 1. Level of premenstrual symptoms was high, with all participants meeting criteria for a PMDs diagnosis (PSTT) (Table 2). Self-reported premenstrual distress was high (>7), whilst ratings of premenstrual coping were moderate (4–5) (SEPQ) (Table 1). Ratings of the use and helpfulness of various strategies for coping with menstrual cycle changes (PMCC) were low. Dyadic adjustment (DAS) and self-silencing (STSS) scores at baseline were comparable to published norms for non-clinical populations, and were in the normal range. HADS depression scores across groups were within the normal range, whilst HADS anxiety scores across groups indicated levels of mild anxiety.

**Table 2:** Mean scores and standard deviations for outcome variables at pre-test, post-test and follow-up by intervention group.

	Couple			One-to-One			Wait-List Control	
Variable	Pre	Post	FUP	Pre	Post	FUP	Pre	Post
	*M (SD)*	*M (SD)*	*M (SD)*	*M (SD)*	*M (SD)*	*M (SD)*	*M (SD)*	*M (SD)*
	(*n* = 28)	(*n* = 19)	(*n* = 15)	(*n* = 30)	(*n* = 23)	(*n* = 21)	(*n* = 25)	(*n* = 21)
***PSTT***								
Emotional Reactivity/Mood	10.94(2.96)	8.67(3.68)	8.0(3.95)	12.52(2.41)	10.10(3.22)	9.38(3.31)	11.33(3.56)	11.30(3.87)
Lack of Energy/Interest	7.72(3.72)	6.14(2.75)	6.20(4.46)	8.02(3.04)	6.22(3.07)	7.43(3.25)	7.78(2.71)	7.65(2.85)
Physical Symptoms	5.97(2.80)	5.19(2.87)	4.40(2.32)	6.89(2.27)	5.55(2.54)	6.24(2.10)	7.50(2.48)	7.30(2.89)
Total Premenstrual Symptoms	24.64(7.96)	20.0(8.12)	18.60(9.10)	27.43(6.32)	21.86(6.97)	23.05(7.26)	26.60(7.30)	26.25(8.48)
***SEPQ***								
Premenstrual Distress	7.73(1.38)	4.00(1.86)	4.03(2.09)	8.07(1.61)	5.15(1.63)	5.71(2.35)	7.27(2.09)	7.14(1.87)
Premenstrual Coping	4.09(1.41)	7.55(1.55)	7.30(2.20)	4.65(2.11)	7.17(1.77)	6.48(1.43)	5.06(2.03)	6.28(2.26)
***PMCC***								
Menstrual Specific Coping	8.75(4.20)	9.13(3.45)	11.53(5.34)	8.87(4.34)	9.61(4.76)	11.48(6.38)	11.12(4.39)	11.33(6.29)
Active Behavioral Coping	5.96(3.97)	10.11(4.84)	8.20(6.02)	5.80(3.53)	7.61(6.02)	6.98(4.63)	6.20(4.54)	6.71(4.36)
Active Cognitive Coping	9.96(5.39)	18.84(8.10)	17.47(10.61)	11.75(5.39)	16.89(9.17)	15.52(8.81)	6.20(4.54)	16.05(9.12)
Avoidance Coping	4.14(2.99)	2.84(3.42)	2.60(1.76)	4.55(3.16)	3.91(3.71)	3.71(2.21)	4.68(3.38)	3.90(3.30)
***DAS***								
Relationship Satisfaction	96.43(15.20)	94.71(24.59)	94.77(21.78)	95.65(11.82)	93.41(22.34)	96.12(18.84)	92.40(15.64)	93.26(22.86)
***STSS***								
Self Silencing	82.52(17.71)	73.73(23.03)	80.20(19.57)	87.47(16.71)	73.30(21.70)	79.71(15.86)	83.62(21.45)	80.10(24.43)
***HADS***								
Depression	6.68(4.47)	3.00(3.14)	3.90(3.23)	6.83(3.99)	4.96(4.25)	5.02(3.42)	7.32(4.31)	5.50(4.75)
Anxiety	9.25(4.41)	5.84(2.93)	6.60(3.94)	10.27(4.30)	7.78(3.25)	7.57(3.26)	10.20(4.64)	9.31(5.48)

### Changes in outcomes measures at post-test by treatment group

The mean scores for all outcome measures at pre-test and post-test for each intervention group are shown in Table 2.

For pre-test to post-test comparisons, a significant time main effect was revealed on all variables, with the exception of relationship adjustment, menstrual specific coping and avoidance coping. Significant reductions were found for total premenstrual symptoms (PSTT) (*F*_1,57_ = 22.16, *p* < .001, *n*^*2*^*p* = .28), as well as for the subscales of emotional reactivity/mood (*F*_**1,57**_ = 21.72, *p* < .001, *n*^*2*^*p* = .28), lack of energy/interest (*F*_1,57_ = 11.40, *p* = .001, *n*^*2*^*p* = .17), and physical symptoms (*F*_1,57_ = 7.33, *p* = .009, *n*^*2*^*p* = .11). Significant reductions were also found for premenstrual distress (SEPQ) (*F*_1,60_ = 95.77, *p* < .001, *n*^*2*^*p* = .62), self–silencing (STSS) (*F*_1,60_ = 15.18, *p* < .001, *n*^*2*^*p* = .20), depression (*F*_1,60_ = 24.03, *p* < .001, *n*^*2*^*p* = .29), and anxiety (HADS) (*F*_1,60_ = 15.22, *p* < .001, *n*^*2*^*p* = .20), with significant improvements over time for premenstrual coping (SEPQ) (*F*_1,60_ = 102.86, *p* < .001, *n*^*2*^*p* = .63), active behavioural coping (PMCC)(*F*_1,60_ = 13.78, *p* < .001, *n*^*2*^*p* = .19) and active cognitive coping (PMCC) (*F*_1,60_ = 31.63, *p* < .001, *n*^*2*^*p* = .35). Main effect differences between intervention groups and interactions were not significant, for lack of energy/interest and physical premenstrual symptoms (PSTT), avoidance coping (PMCC), relationship satisfaction (DAS), self-silencing (STSS), depression and anxiety (HADS). There was a statistically significant interaction between intervention group and time for total premenstrual symptoms (*F*_2,57_ = 4.74, *p* = .01, *n*^*2*^*p* = .14) and emotional reactivity/mood (*F*_2,57_ = 5.35, *p* = .01, *n*^*2*^*p* = .16) (PSTT); premenstrual distress (*F*_2,60_ = 14.60, *p* < .001, *n*^*2*^*p* = .33) and premenstrual coping (*F*_2,60_ = 6.64, *p* = .002, *n*^*2*^*p* = .18) (SEPQ); and active cognitive coping (*F*_2,60_ = 6.12, *p* = .004, *n*^*2*^*p* = .17) (PMCC).

Post-hoc ANOVA tested significant interaction effects. Post-intervention, total premenstrual symptoms (PSTT) were significantly higher in the wait-list control group compared to the couple intervention (*MD* = 6.25, *SE* = 2.55, *p* = .045) but not the one-to-one group, with no significant difference between the two active intervention groups. A statistically significant effect of time, indicating lower total premenstrual symptoms at post-intervention, was found for the couple (*F*_1,17_ = 8.004, *p* = .011) and one-to-one groups (*F*_1,21_ = 24.11, *p* < .001) but not the wait-list control group. Premenstrual distress (SEPQ) was significantly higher post-intervention in the wait-list control group (*MD* = 3.14, *SE* = 0.57, *p* < .001) compared to the couple intervention and the one-to-one groups (*MD* = 1.99, *SE* = 0.54, *p* = .001), but was not significantly different between the two active treatment groups. There was a statistically significant effect of time, with lower premenstrual distress at post-intervention for the couple (*F*_1,18_ = 53.78, *p* < .001) and one-to-one groups (*F*_1,22_ = 50.76, *p* < .001), but not the wait-list control group. For emotional reactivity/mood symptoms (PSTT), premenstrual coping (SEPQ), and active cognitive coping (PMCC), differences between intervention groups were not significant. There was a statistically significant effect of time, with lower emotional reactivity/mood symptoms (PSTT) at post-intervention for the couple (*F*_1,17_ = 46.70, *p* = .005) and one-to-one groups (*F*_1,21_ = 65.05, *p* < .001) but not the wait-list control group. There was a significant effect of time with increases in premenstrual coping (SEPQ) at post-test for all groups: couple (*F*_1,18_ = 47.55, *p* < .001), one-to-one (*F*_1,22_ = 36.43, *p* < .001), and wait-list control (*F*_1,20_ = 17.50, *p* < .001). Active cognitive coping was significantly increased at post-test for the couple (*F*_1,18_ = 29.23, *p* < .001) and one-to-one groups (*F*_1,22_ = 13.33, *p* = .001) (PMCC).

Participants reported no adverse outcomes to the research team or the University Human Research Ethics Committee, and no participants withdrew consent during the study.

### Follow-up comparisons

The mean scores for all outcome measures for the couple and one-to-one treatment groups at follow-up are shown in Table 2. For post-intervention to follow-up comparisons, no significant time or interaction effects were revealed on any of the measured variables indicating that improvements in scores were maintained during the follow-up period.

### Change in PMDs status

Chi-squared analyses were used to assess differences in PMDs casesness over time for each treatment group, and group differences at post-test and follow-up (PSTT) (Table 3). Significantly fewer women met PMDs caseness criteria at post-test compared to baseline in all treatment groups: *X*^*2*^_*(1*,*19*)_ = 8.07, *p =* 0.004 for the couple group; *X*^*2*^_*(1*,*23*)_ = 5.54, *p =* 0.019 for the one-to-one group; and *X*^*2*^_*(1*,*21*)_ = 5.09, *p =* 0.024 for the wait-list control group. The proportion of women meeting PMDs caseness criteria at follow-up did not significantly differ from post-test levels for the couple and one-to-one intervention groups. There were no significant differences in PMDs casesness rates between the intervention groups at post-test, *X*^*2*^_*(2*,*63*)_ = 0.55, *p =* 0.76, or at follow-up, *X*^*2*^_*(1*,*36*)_ = 0.01, *p =* 0.94.

**Table 3:** Frequency of PMDs casesness at pre-test, post-test and follow-up by intervention group (PSTT).

	Couple		One-to-One		Wait-list Control	
Time / Case	*n*	*%*	*n*	*%*	*n*	*%*
Pre-treatment						
PMDs	28	100	30	100	25	100
Non-PMDs	-	-	-	-	-	-
Post-treatment						
PMDs	14	73.7	19	82.6	17	81.0
Non-PMDs	5	26.3	4	17.4	4	19.0
Follow-up						
PMDs	13	86.7	18	85.7	-	-
Non-PMDs	2	13.3	3	14.3	-	-

### Subjective experience of premenstrual change

#### Emotional reactivity pre-post intervention

In the open ended survey responses and interviews, the majority of participants reported feeling differently about those around them in terms of increased negative emotional reactivity premenstrually, including increased anger, irritation, and anxiety (Table 4). In the open-ended surveys, post-intervention negative emotional reactivity was reported by a smaller proportion of women within the one-to-one and couple conditions (reduction of 61% and 64% respectively), compared to the wait list control (reduction of 35%) (Table 4). This was manifested by a reduction in both intensity and frequency of negative emotion.

**Table 4:** Pre-post intervention: Premenstrual changes in emotional reactivity (open ended-survey responses).

Pre-intervention			Post Intervention		
Feeling angry, irritable and oversensitive			Feeling angry and irritable with reduced intensity		
Group	%	*N*	Group	%	*N*
WLC	64	16	WLC	29	6
One-to-one	70	21	One-to-one	9	2
Couple	75	21	Couple	11	2
I can’t stand everyone. I have a very negative outlook during this time and people’s negative characteristics/flaws are exacerbated which infuriates me. Things which are normally minor get to me and make me super angry (WLC).			I still hate my husband and now believe that my anger is probably justified and I wonder if those feelings are really a true indication of how I feel about him (WLC).		
People annoy me more and I can’t stand to be around children (WLC).			I feel when premenstrual that I want to be straightforward but sometimes it comes across as blunt; I have now become better at holding rudeness from my tone and feel this is an achievement (WLC).		
I’m more sensitive and more easily offended or upset (WLC).			I can feel the signs–I’m touchy, cranky, irrational,—so I try to take a step back and start my breathing techniques. I tell myself everything is OK, just the way it is and remind myself how important my family and friends are to me (one to one).		
I feel everyone is against me or they are not trying to understand (one-to-one).			I still experience irritability, but I am not a big ball of screaming rage any more (one to one)		
I am less loving as most things annoy me about people–especially those close to me (one to one).			I still get that irritation, that frustration. But it’s probably over a shorter period of time, and maybe it doesn’t happen as often (one to one)		
I get more irritated and am more likely to be sarcastic and rush people off. I feel a bit more distant from others as well (one-to-one).			I don’t experience “the highs and lows to the same extent. I’ve been a lot more stable recently (couple).		
I get very angry. Very irritable and that builds up to being very angry. I don’t want the irritation of having to talk to other people, let alone deal with requests and dealing with the needs of small children (one-to-one).			I don’t experience “the highs and lows to the same extent. I’ve been a lot more stable recently (couple).		
I find people annoying and any minor transgressions or habits easily upset me or provoke my anger. I tolerate them less (couple).			I still get frustrated but I can control it better (couple).		
I would prefer to be alone so feel resentful/angry when people put demands on me that I see as unnecessary or I don’t want to participate in (couple).			I still get irritated but am now able to keep it in check by taking a deep breath or telling myself it wasn’t worth the blood pressure rise (couple).		
I get jealous and paranoid “everybody hates me” syndrome! (couple).					

#### Feeling fat and ugly: Women’s feelings about the premenstrual body

The majority of participants reported negative feelings towards their bodies when they were premenstrual, describing themselves as “fat”, “ugly”, “frumpy”, “sluggish”, and “unattractive”. Some of these negative feelings were associated with embodied changes such as bloating and painful breasts, but women also described themselves as more “self-conscious” and “self-critical” of their bodies during the premenstrual phase of the cycle. Post-intervention, there was a greater reduction in such reports in open ended surveys in the couple condition (63%) and one-to-one condition (47%), than in the wait list control (25%) (Table 5). This was manifested by greater acceptance and understanding of embodied change reported by the majority of participants in the one-to-one (68%) and couple (72%) conditions, and a minority of the wait list control (5%).

**Table 5:** Premenstrual changes in women’s feelings about their bodies (open ended-survey responses).

Pre-intervention			Post Intervention		
**Feeling fat and ugly: Negative perceptions of the body**			**Feeling fat and ugly: Negative perceptions of the body**		
**Group**	**%**	***N***	**Group**	**%**	***N***
WLC	84	21	WLC	59	13
One-to-one	83	25	One-to-one	34	8
Couple	78	22	Couple	15	3
I suppose I just feel ugly, so my dissatisfaction is with my overall appearance. I usually experience this very particular kind of irritation where I feel irrationally fat and hate everything about my figure and clothes, when I experience this I know my period will start in a couple of days (WLC).			I feel bloated and it makes me feel like my body take up more space. I also feel betrayed by my body (WLC).		
Yes. I feel unattractive. I know I still look the same–it is all in my mind but that doesn’t make me feel any better. I feel fat. I also will dress differently at that time of the month (WLC).			I feel disappointed about my body. I feel bloated and tired so I never look good in anything I wear (WLC).		
Yes I hate it. I feel like an elephant, very unattractive and I over compensate by putting pressure on my partner. I don’t like looking in the mirror (one-to-one).			Yes absolutely. I feel like a whale and hate my body during this time. It makes me hide my body and I wear bigger things to try to do this (WLC).		
I like my body less than usual, I feel fat and bloated and am more aware of my flaws. I am more self-conscious about my belly and thighs, which feel flabby, and am less confident about my appearance and general body shape (one-to-one).			I always feel fat and yuck. I think because I’m bloated I just feel fat and ugly. I don’t like my body when I’m premenstrual (one-to-one).		
More critical of bumps. Not as good as it once was. Just fat and ugly (one-to-one).			I still feel bloated and heavy which makes me feel uncomfortable. I try not to look at my body when like this to avoid telling myself how bad I look even though it’s not that much different to the rest of the month (one-to-one).		
I feel more self-conscious–I feel fat and ugly (couple).			Feel fat and bloated, more self-conscious (couple).		
I see all faults and feel that they are larger than they are (ie stomach, thighs) to the point that I can’t stand to look at myself (couple).			Feel fat and ugly (couple).		
**Acceptance and understanding of embodied change**			**Acceptance and understanding of embodied change**		
**Group**	**%**	***N***	**Group**	**%**	***N***
WLC	0		WLC	5	1
One-to-one	0		One-to-one	63	15
Couple	0		Couple	72	16
			Yes, after realising the pattern, I feel better about myself as I feel I am helping myself overcome bad feelings. I start to love myself at that time of the month, instead of hating myself (WLC).		
			Yes, I don’t get as worried when I feel/look a bit heavier and sometimes I don’t even notice it anymore (that is, sometimes I don’t feel/look heavier) (one-to-one).		
			Yes, I don’t feel so badly about being bloated or slightly bigger in that time, in fact I hardly notice it now (one-to-one).		
			I notice changes in my body (feel bloated in stomach and thighs) but tell myself ‘this is just because I am premenstrual, it will be better in a few days’. And my partner reassures me about my appearance. (one-to-one).		
			I am less annoyed by the process and symptoms and able to relax and accept it (one-to-one).		
			I understand now that I need to take it easy as my body is under stress. There is nothing wrong or bad about my body (one-to-one).		
			What used to bother me before–bloating and not liking what I saw in the mirror, now doesn’t seem to bother me as much, I do not dwell on it as much as I did before (couple).		
			I’ve accepted the physical changes my body undergoes, even though I dislike it, and am easier on myself (ie. Not thinking I’m fat and ugly all the time) (couple).		
			I can now link the brain behaviour to the body chemistry, so can be more understanding (couple).		

#### Intimate relationship difficulties and support pre-post intervention

The majority of women reported exacerbation of relationship tension and intolerance towards their partner in the premenstrual phase of the cycle (Table 6), using a short-fuse or pressure cooker metaphor. In the open ended survey responses, there was an 18% reduction in reports of intimate relationship difficulties within the couple conditions, compared to a 10% increase in the wait list control (10%) and 5% increase in the one-to-one condition (5%) (Table 6). Post-intervention, women reported that they were less likely to “lose control” when expressing their feelings, had increased awareness of the potential for relationship conflict, or described relationship tension as less problematic.

**Table 6:** Pre-post intervention: Premenstrual relationship difficulties and support (open ended-survey responses).

Pre-intervention			Post Intervention		
Intimate relationship difficulties: Short fuse and pressure cooker			Intimate relationship difficulties: Short fuse and pressure cooker		
Group	%	*N*	Group	%	*N*
WLC	56	14	WLC	66	14
One-to-one	43	13	One-to-one	47	11
Couple	60	17	Couple	42	8
Oh Yes! I nearly always want to leave the relationship due to finding fault with him, me, us! Or leave my home and go far away. I did this many times in my 20’s before I understood what was going on with me (WLC).			I am quicker to bring up issues when premenstrual. Things that don’t bother me normally do bother me and this affects my relationship (WLC).		
Underlying issues in the relationship tend to come up during my PMS time, as I feel irritable and have low tolerance. We are more likely to fight at this time (WLC).			If we have a misunderstanding or poor communication I get frustrated, that it seems we are not on the same page, and I feel impatient and will cause a breakdown in communication (WLC).		
if he makes a certain comment or if we’re driving and he takes the wrong turn I’ll make more of an issue of it then I would normally (WLC).			I think because I am more aware of a tendency to fight with my partner now, I try not to bring up issues around that time (WLC).		
We bicker more as I pick more on things my partner does. He gets in trouble for breathing too heavy or tapping or jiggling I get so irritable (one-to-one).			The problems I have in my relationships always seem too hard to deal with when I’m premenstrual and I always feel like it’s easier to give up and walk away (one-to-one).		
My husband and I have our strongest disagreements and there is a lot of anger on my part over what are essentially small things, but which I see as being vital to the smooth running of the evening routine with our daughters. The arguments are invariably in the evening (One-to one).			I still get angry at my husband as he doesn’t understand my PMS. However I think it’s the PMS makes me feel this, as he does try and avoid the issues at this time (one-to-one).		
I keep things to myself more when I’m not pre-menstrual and just get on with it and then I just explode at my husband just before I’ve got my period (one-to-one).			Yes, this is still the case, as our differences seem to acute and I have no desire to be intimate. However, I make a point of not making any major decisions during this time, and I try to hold my tongue or remain calm with my partner (couple).		
All our fights–with my husband and I occur during my PMS. I know it is an issue and I know during PMS what is going on but I can’t control my feelings despite that (couple).			I get depressed, withdrawn and keep things to myself. The bothers husband, who can see I’m not well but wouldn’t discuss with him (couple).		
I am more likely to snap if something has been building up for a while. I can be affected a lot more by any problems. I can get a bit crazy and even feel like I can’t cope to the point where I want to break-up with my fiancé. I also become quite depressed if my sexual and emotional needs aren’t being met at a time when I sometimes have increased desires. I feel I can’t express my needs/feelings clearly and so either say nothing or explode (couple).			We still fight, but when it happens it’s much less intense (couple).		
I feel Less loved (couple).			PMS is just a build-up of little things that have pissed me off and it comes to the point where I’ve had enough, and would really like to separate—but we can’t really (couple).		

Increased partner awareness and understanding of the premenstrual phase of the cycle, associated with greater support, was reported in 19% of the wait list control, 39% of participants in the one-to-one condition, and 84% of those in the couple condition post-intervention (Table 7), in the open-ended survey responses. This was associated with reports of an improved relationship with a woman’s intimate partner, in 57% of participants in the couple condition, 26% in the one-to-one condition, and 5% of the wait list control (Table 7). These improvements included resolution of relationship difficulties, greater couple communication, and greater closeness.

**Table 7:** Positive changes to intimate relationships post-intervention (open ended-survey responses).

Increased partner support and understanding			Improved relationship		
Group	%	*N*	Group	%	*N*
WLC	19	4	WLC	5	1
One-to-one	39	9	One-to-one	26	6
Couple	84	16	Couple	57	11
I keep the survey that I am doing on the computer desk so I do it every night, and I think he sort of has a bit of a check of that, and a bit of a read and says “Okay, this is it,” which is a good thing for him to see instead of just not being nice about it. Like I think he’s actually seen it is something (WLC).			A better relationship. A better me! (WLC).		
I think about breaking up because I can’t stand myself when I am like this and neither can he. However–he has started to understand that it is hormonal and doesn’t take my moodiness and outbursts so seriously (WLC).			I think it has improved my relationship with my now fiancé. I have been more open when I am feeling depressed and he gives me lots of hugs. I think I have also identified patterns of negative behaviour which occur PMS or not and that I need to work on this not just PMS time but all the time (one to one).		
I think my husband has become 100% more understanding after reading the surveys and after knowing how PMS made me feel. It was a real thing, not me being “crazy” (WLC).			My partner and I are closer. We communicate more. We enjoy activities together more often. He is more supportive when I am going through PMS (one-to-one).		
Now that my partner has a better understanding of what’s happening and I’m more aware of my actions, we work together in trying to argue less and it seems to be having a positive effect (one-to-one).			Most of our relationship issues related to my feelings during PMS have now resolved. My partner is much more supportive now that he knows what I’m going through (couple).		
I tell my partner that my period is coming and I am having a few dark days. He understands now and knows that I need a few more hugs (one-to-one).			We are now able to openly discuss our issues and so they are now shared issues. Brought us closer together (couple).		
Issues would still come up but I was less likely to fly off the handle and get crazy about it. I’d be more likely to just say what I needed to say and let it go or have a rational discussion about it instead of just yelling or sulking or whatever (one-to-one).			My partner and I have benefitted so much from this study. It has allowed me to be introspective without being judged and learn more about myself, my PMS and my relationship. My husband and I communicate better and I haven’t had an angry outburst with him since the study. Thanks!! (couple).		
I find my partner is even more sympathetic towards me in my PMS phase. The study has allowed him a greater insight into how PMS affects me–so now when I’m feeling angry/stressed/irritable, we talk about PMS and he tries as best as he can to make me feel better (couple).					
Now my partner understands the depths of despair PMS can create (ie that it is an actual thing ALOT OF WOMEN GO THROUGH) he tries to be more helpful and understanding in those times. I try to be calmer and less needy, taking more time for myself (couple).					
Most of our relationship issues related to my feelings during PMS have now resolved. My partner is much more supportive now that he knows what I’m going through (couple).					

#### Positive consequences of the intervention: Increased awareness, communication and coping

Participants reported a number of positive consequences of taking part in the intervention study, in addition to relationship improvement (Table 8). The majority of participants across conditions reported increased awareness and understanding of premenstrual change, associated with greater control over emotions, and less anger or irritation towards others. In the open ended survey responses, women in the two active intervention conditions were more likely to report improved communication and asking for help following participation in the study (68% couple condition, 65% one-to-one), than women in the wait list control (9.5%).This was associated with reports of increased knowledge and comfort in discussing premenstrual change, learning specific communication skills associated with expressing premenstrual needs, and awareness of the importance of communication in relationships, and feeling “understood” by others.

**Table 8:** Positive consequences of the intervention: Self-care, communication and coping (open ended-survey responses).

Increased awareness and understanding of premenstrual change			Improved communication and asking for help			Improved coping skills			Self-care and coping to deal with embodied change		
Group	%	*N*	Group	%	*N*	Group	%	*N*	Group	%	*N*
WLC	57	12	WLC	9.5	2	WLC	33	7	WLC	9	2
One-to-one	16	16	One-to-one	65	15	One-to-one	100	25	One-to-one	26	12
Couple	57	11	Couple	68	13	Couple	100	26	Couple	58	10
*I can see that my mood changes when premenstrual and I need to try not to take this out on others*. I still get very angry and irritable but try to remember that this is due to PMS (WLC).			I feel very aware of my feelings and try hard to be happy around family, or *tell them straight away if I’m feeling bad*, *then they help me* (WLC).			I found it really interesting to keep the daily diaries, and reading through them each week. I found it great to talk to my partner about my PMT–to find out his point of view on my “week”! *It has made me put strategies in place to deal with my PMT* (WLC).			I’m eating more healthily now but still have cravings from time to time, but I feel my body react better when PMS (WLC).		
*Seeing patterns in behaviour and mood changes–makes me aware* and I try to change or lessen moody behaviour (WLC).			My husband and I have an understanding where *I let him know it is “pmt” week (he can usually pick it*!*)* and he is very understanding! (WLC).			Becoming more aware of my worsening moods has led me to be able to *talk myself out of those moods and hence reduce my PMS symptoms* (WLC).			Only that I need to look after my body throughout my cycle. So yoga– 2 times/week; Epsom salts baths– 1 every 1–2 weeks; eating more protein at lunch; drinking less coffee (one-to-one).		
I’ve sort of seen a trend. *I’ve sort of learnt now what to expect*, *so it’s*, *like it’s not so much unknown now*. So I think that’s how I’m dealing with it a bit better as well (WLC).			*Yes I feel that they understand me now*. *I talked to everyone (friends and family)* and feel more comfortable being myself and socialising when premenstrual (one-to-one).			I was made more aware of my PMS and *taught myself to manage it* (WLC).			I have changed to a much healthier lifestyle so now when I look in the mirror I see that progress I’ve made rather than hating my body. I am more accepting of monthly changes and remind myself that it’s only for a short time (one-to-one).		
*I used to think that others changed at that time of the month*. *Now I can recognise that it’s my perceptions that change* so I am careful not to attack/criticise them (one-to-one).			*I find it easy to say “I’m premenstrual” and talk about how I’m feeling about situations or events compared to how I’m feeling when I’m not premenstrual* (one-to-one).			My life had improved across the board. After years of horrid PMS and feeling that no matter what I tried it never consistently worked, *I now feel like I can cope*, *everyday and if I have a bad day*, *I no longer feel out of control or that it’s the end of the world*, *such a relief*! (one-to-one).			I feel I can now better deal with being premenstrual. I take better care of my body and listen to my needs (one-to-one).		
*I try to remain very conscious that I am more sensitive–not that they are more annoying*! (one-to-one).			*I think I just communicate it more effectively now*. *So I’m just better able to say what I want without the agitated emotional mannerisms*. *I’m able to express just very clearly whether I do want to be touched or I don’t and he’s fine with whatever*. (one-to-one).			*I’ve made time for myself each week*. *Lived each day at a time to try avoid being overwhelmed*. *Been less ‘hard on myself’ Tried to stay away from conflict where possible* (one to one).			Yes, I know that if I eat the right food with treats in moderation I can cope better. Increasing my exercise regime helps me to think more clearly and react more positively to every day challenges (couple).		
I am learning to be more tolerant. *They are not the issue it’s the PMS* (one-to-one).			*I now feel I can tell my partner what is happening before I snap at him (or take things out on him*) (couple).			I can feel the signs–I’m touchy, cranky, irrational,—*so I try to take a step back and start my breathing techniques*. *I tell myself everything is OK*, *just the way it is and remind myself how important my family and friends are to me* (one to one)			I am not as extreme in my self-criticism–I still experience bloating and feeling not as good looking, but I get pampering things done, wear more comfortable clothes, accept myself and tell myself it will change shortly (couple).		
*I didn’t realise there was a pattern to premenstrual night terrors until I was keeping those journals and I went*, *“Oh*, *my God*” (couple).			I know and understand that *by talking to my partner things are not as grim as I thought*. I appreciate him more (couple).			When I know I am in the PMS phase of my cycle *I constantly try to take each thing that comes my way not letting it make me more upset and move into something calmer—ie do some relaxing*, *interesting thing for myself*, ask for help from partner (couple).			I feel more in-tune to the symptoms of my body. I’m able to help myself for a change. I’m able to respond appropriately to the changes of my body and more (couple).		
*I am more aware of my criticism so I have more space and am more tolerant*. I realise it is PMS thinking (couple).			It’s not as bad as before, especially since *we have been working on our communication during the last few months*. This means that there aren’t so many pressing issues that get out of hand at PMS time. We still have some work to do though (couple).			I’m better able to cope with mood changes during PMS (more aware, can ask for help, reassurance, positive thinking) (couple).					
Yes–*I feel that I can recognise stressors and why I suddenly arc up*. I feel I have been given some tools (couple).						I now analyse my thoughts, feelings, and actions more, and I’m now able to catch myself when my thoughts and feelings are negative or not conducive to my well-being. So I’m able to use the strategies I learned in the study to avoid having my feelings get on top of me (couple).					

The development of active coping skills to deal with premenstrual changes was reported by all of the women in the two active conditions, and a third of the women in the wait list control group. This included self-talk to reduce premenstrual negative moods, changing perceptions of premenstrual emotion, and recognition of premenstrual needs. Awareness of cyclical changes facilitated self-care, including avoidance of conflict, active engagement in self-care. These self-care and coping strategies were reported to have a beneficial effect on women’s moods, and their ability to control the expression of negative emotion. There was also a positive benefit for women’s experience of embodied change during the premenstrual phase of the cycle, with over half of the women in the active conditions associating active coping strategies with “looking after my body” or “feeling better about my body” post-intervention, in comparison to less than a tenth of women in the wait list control group (Table 8).

## Discussion

This study examined the relative efficacy of a couple CBT for PMDs in comparison with a one-to-one CBT therapy, and a wait list control group. There were no pre-intervention differences between the three groups, suggesting that they were comparable in meeting the criteria for PMDs, experiencing high premenstrual distress, moderate-low levels of premenstrual coping, and mild anxiety. Levels of depression, relationship adjustment and self-silencing were in the normal range across groups. The finding of significant differences across time when the groups are collapsed (ignoring the intervention group assignment), confirms previous reports [10, 13, 14] that taking part in an intervention for PMDs can reduce psychological premenstrual symptoms and premenstrual distress [15, 16, 18, 20, 72, 73]. We also found significant reductions in depression, anxiety and self-silencing, and improvements in premenstrual coping, suggesting that taking part in the intervention also had a positive impact on women’s psychological wellbeing, her relationship communication strategies and on their ability to address premenstrual change.

In a review of CBT interventions for PMDs, Lustyk and colleagues [10] report that existing RCTs report a main effect of time, but not a statistically significant pre-post difference between intervention groups [15, 16, 18, 19, 73]. Two meta-analyses [13, 14] reported a small non-significant effect size for behavioural symptoms, and a medium effect size for mood, the magnitude of which was “not satisfactory” [14]. In contrast, we found that post-intervention women in the two active CBT conditions reported significantly lower total premenstrual symptoms, lower emotional reactivity/mood, lower premenstrual distress, and higher cognitive coping than women in the wait list control. This suggests that that a CBT intervention for PMDs, in both a one-to-one and couple modality, is effective in reducing premenstrual symptoms, premenstrual distress and premenstrual emotional reactivity, as well as in improving premenstrual coping, at post-intervention, with improvements maintained at follow-up. The absence of post-intervention change in these variables in the wait list control group suggests that positive effects were due to the CBT intervention, rather than to the effects of time, or to taking part in a PMDs intervention study. These findings also suggest that the addition of a woman’s partner to the intervention is not having an effect on her premenstrual symptoms, premenstrual distress or cognitive coping.

The finding of a main effect of time for depression, anxiety, self-silencing, physical premenstrual symptoms and lack of energy/interest, but no post-intervention group differences between groups in these variables, suggests that positive changes were associated with time, individual differences between women, or taking part in the study. Self-monitoring has been shown to be effective in the reduction of premenstrual symptomatology [74], due to increased awareness of the relationship between moods and stressful situations across the menstrual cycle [75], and giving the woman a sense of control, as well as helping her to educate her family [4]. This was confirmed by the qualitative accounts of increased awareness and understanding of premenstrual change post-intervention in the majority of participants in the wait-list control group, attributed to the self-monitoring of premenstrual mood prior to study entry, as well as completion of pre-post surveys.

The qualitative data provides insight into the subjective experience and potential mechanism of changes resulting from participation in the intervention study. Pre-intervention, the majority of women reported feeling angry, irritable and oversensitive, which is central to clinical definitions of PMDs [3], and has been reported in previous qualitative PMDs research [7, 76, 77]. There was a substantial reduction in such reports post-intervention, most markedly in the two active conditions, suggesting a reduction in both intensity and frequency of negative premenstrual emotional reactivity, which confirms the findings of the quantitative outcome measures (PSTT). Increased awareness and understanding of premenstrual change post-intervention was one explanation for these reductions in reactivity, reported by the majority of women across conditions. This confirms previous reports of a reduction in self-pathologisation [48], as well as the use of anticipatory coping strategies [25], following the development of awareness of patterns of premenstrual change. The majority of women in the two active conditions, and a minority of women in the wait-list control, also reported increased communication and help-seeking, as well as the development of coping skills, associated with reductions in premenstrual reactivity and distress. These skills are core ingredients of the CBT intervention [18, 22], and demonstrate the mechanisms of its effectiveness, through changing patterns of thinking, communication, coping and self-care.

Previous research has reported that women’s bodies are often experienced as ‘out of control’ during the premenstrual phase of the cycle [7], and can become the object of critical self-scrutiny [31, 38, 39], with the attribution of premenstrual changes to ‘raging hormones’ leading women to take up the subject position of the ‘monstrous feminine’ [12]. In the present study, the majority of women described embodied self-loathing and feelings of unattractiveness premenstrually, primarily associated with perceptions of bloatedness and weight gain. Previous research has reported that fluctuations in weight preoccupation across the menstrual cycle are influenced primarily by post-ovulation emotional eating, rather than ovarian hormones [78]. However, the psycho-social meaning of feeling ‘fat’, associated with failing to live up to idealised constructions of feminine bodies as slim and in control [38, 79], is arguably central to women’s dislike of the premenstrual body. These meanings can be challenged within a CBT intervention, as reflected in the increased understanding and acceptance of embodied change reported by the majority of women in the active conditions in the present study, associated with marked decreases in negative conceptualisations of the premenstrual body. Women in the two CBT conditions were also more likely to report engaging in self-care and coping strategies to deal with embodied change premenstrually, most notably in the couple condition. These findings suggest that self-perception of the body, rather than simple increase in weight or bloating, are central to women’s feelings of negative premenstrual embodiment. There is a dearth of research on women’s experiences of weight gain [78] or embodiment premenstrually; previous studies examining the efficacy of CBT for PMDs have not examined women’s feelings about their bodies as outcome variables. The strength and uniformity of women’s negative reports in the present study, and the positive changes post-intervention in the active conditions, suggest that this is an area worthy of further scrutiny, as women’s negative perceptions of their bodies are strongly associated with negative perceptions of the self [38, 80]. This suggests that increased acceptance of the premenstrual body can positively impact on women’s acceptance of the premenstrual self, and of premenstrual change.

Whilst previous research has reported that women who report PMDs also report higher levels of relationship dissatisfaction or difficulties [26–31], this was not found in the present study, where levels of relationship adjustment as measured by the DAS were in the normal range. Equally, the finding that no change in relationship adjustment as measured by the DAS was reported pre-post intervention, indicates that taking part in the study did not significantly impact upon global relationship adjustment, within or between groups. However, in the qualitative open-ended survey items women reported exacerbation of relationship tensions, or greater irritation towards their partner premensturally, confirming previous research of altered perceptions of family and relationship stresses [24, 25], and negative evaluation of intimate relationships [32, 33] in the premenstrual phase of the cycle. These relationship tensions were still present post-intervention to the same or a greater extent for the wait-list and one-to-one conditions, in contrast to a reduction in the couple condition. The finding that women in the couple and one-to-one condition were more likely to report increased partner understanding and improved relationship post-intervention, with women in the couple condition reporting these improvements to a greater degree, confirms previous reports that CBT interventions can have a positive effect on relationships [18, 47]. However, it suggests that couple based CBT interventions can have a greater positive impact upon perceptions of relationship context, reinforcing the plea for couples therapy as an appropriate form of intervention for PMDs [45], and suggesting that partner involvement can increase relational support [30].

This study had a number of strengths and limitations. The strengths were the use of daily diary measures and a standardised screening instrument to establish PMDs criteria during the recruitment phase; comparison of two active treatments with a wait list control within a randomised controlled trial design; and the use of a range of qualitative and quantitative measures allowing for the extent of change within and between groups to be evaluated with repeated measures ANOVA, alongside women’s subjective experience of change. Whilst the rate of attrition post-intervention was a limitation, resulting in a small sample size, attrition was lower than that reported in previous CBT RCTs for PMDs [10], and the groups were of sufficient size to allow for adequate statistical power to detect a significant difference between intervention groups. A limitation was the absence of daily diary ratings throughout the study, which would have allowed for measurement of ongoing change. This was not possible due to participant survey fatigue, with completion of daily diary ratings pre-randomisation reported to be the only negative aspect of taking part in the study. Whilst partners were included in the couple condition, no evaluation of the perspectives of partners across conditions was included. This would strengthen the design of future couple interventions for PMDs, allowing for evaluation of the impact of intervention on partners, and possible changes in their perspective on women’s PMDs. The majority of women who took part in this study were in heterosexual relationships. However, the one-to-one intervention used in the study has been found to be efficacious with women who are single and in lesbian relationships in previous research [56, 73], suggesting it has efficacy beyond women in a heterosexual couple.

### Conclusion

In conclusion, the findings of this study suggest whilst taking part in an intervention study for PMDs can have a beneficial impact in reducing premenstrual symptoms and premenstrual distress, and in improving premenstrual coping, active CBT is more beneficial than a wait-list control. Therefore, whilst daily monitoring may increase awareness of premenstrual change, the active ingredients of a women-centred cognitive behavioural intervention are more efficacious in reducing symptomatology and increasing a woman’s sense of control of her premenstrual moods, as well as her ability to cope with psychological and embodied premenstrual change. This can have positive consequences for a woman’s perception of her relationship in the premenstrual phase of the cycle, with couple-based interventions having a greater impact in this regard.

Couple based CBT interventions may have a greater positive impact upon behavioural coping and perceptions of relationship context and support. This suggests that CBT should be available for women reporting moderate-severe PMDs, with couple-based CBT offering additional benefits to a one-to-one modality. Whilst CBT may be more costly than bio-medical treatments in the short term, as it requires more intensive clinician time and trained personnel [10], the finding of a benefit over a relatively short time period suggests that long term costs may be lower than the ongoing medical treatment that is recommended for severe PMDs [4]. Equally, a psychological intervention may be preferable to women who experience side effects of medical treatments for PMDs and who withdraw from treatment as a result [4, 9]. Finally, the demonstration of efficacy of a CBT intervention for PMDs confirms the viewpoint that premenstrual distress is not simply a phenomenon located in women’s physiology, but that it is closely tied to psycho-social factors [12, 39], including a woman’s perception of ‘PMS’ and the premenstrual self, her relationship context, and her means of coping with embodied or psychological change throughout the whole cycle. These findings support the argument that CBT can have a preventative effect for PMDs [10], by preventing the premenstrual increases in negative symptomatology or changing the way women come to relate to and report those symptoms. The inclusion of a psycho-social model of premenstrual change in health education for young women, and more broadly in education about adult women’s reproductive health, may go some way to increasing understanding of women’s cyclical mood change, as well as increasing women’s increasing agency and coping, thus reducing rates of premenstrual distress in the community. This will have positive consequences for women and their families, as well as for women’s social and economic functioning.

## Supporting information

S1 Checklist CONSORT 2010 checklist. (DOC)

S1 Protocol Study protocol. (PDF)

S1 Dataset Study data set file. (SAV)
