# Supplementary material for: Evaluation of the relative efficacy of a couple cognitive-behaviour therapy (CBT) for Premenstrual Disorders (PMDs), in comparison to one-to-one CBT and a wait list control: A randomized controlled trial
Source: PLoS One. 2017 Apr 18;12(4):e0175068. doi: 10.1371/journal.pone.0175068 (PMC5395168; doi:10.1371/journal.pone.0175068)
Supplement: S1 Protocol — (PDF) [file pone.0175068.s002.pdf]

National Ethics Application Form
Version 2008 - V2.0

Proposal title: **Evaluation of the relative efficacy and mechanisms of a couples-based intervention for Premenstrual Syndrome through a randomised control trial using mixed methods**

For submission to:
University of Western Sydney Human Research Ethics Committee (EC00314)

Name: **Dr Janette Perz**

Address: **University of Western Sydney, Locked Bag 1797 Penrith South DC NSW 1797**

Contact: (Bus) **02 9772 6512**
(AH) **-**
(Mob) **-**
(Fax) **-**

Proposal status: **Complete**

Proposal description:

The aims of this project are two fold. Firstly, to evaluate the relative efficacy of a brief couple-based PMS intervention, in comparison to a one-to-one PMS intervention, and a self-monitoring control group. The second aim is to identify the effective mechanisms of PMS interventions through process change evaluation.
A RCT, and triangulation of methods will be employed to meet the first aim of the study. This allows for the comparison of active treatment with self-monitoring, and the comparison of two active treatments. Process evaluation will also be conducted by the psychologist administering the interventions and the participants to examine mechanisms of treatment effect.
Women will be randomised to one of three conditions, using disproportionate stratified random sampling. The first condition includes a one-to-one PMS intervention that uses a combination of narrative and cognitive-behavioural techniques (Ussher, Hunter et al. 2002). The second condition is a couple-based PMS intervention, developed by the current project team, supported by a UWS internal grant, differs from the above one-to-one PMS intervention primarily in terms of modality (couple versus individual), with the addition of Couples Dialogue techniques (Hendrix 1990. Three monthly 90-minute sessions of therapy and a 2-month booster session will be conducted by a registered clinical psychologist. The third is a control group. Women in this condition will complete daily PMS diaries, as well as evaluation measures at baseline, and 5 months later, to mirror the pre-post evaluation of the treatment conditions.
Outcome measures include the Hospital Anxiety and Depression Scale (Zigmond and Snaith 1983); a daily rating of severity of problems form (Endicott and Harrison 1990); the Dyadic Adjustment Scale (DAS) (Spanier 1976); subjective evaluation of PMS questionnaire (Ussher and Perz 2006); affectional expression subscale of the DAS, clinical observation of couples, and self-report; experience of intervention (will be evaluated post-treatment, using Likert scales and open ended questions.
Semi-structured Narrative Interviews, previously used to examine moderate-severe PMS in both the UK and Australia, will also be employed (Ussher 2003; Perz and Ussher 2006; Ussher, Perz et al. 2007). Post-intervention, a second interview will be conducted, with the same questions being addressed, with a focus on how the intervention has impacted in each of these areas. Interviews will be audio-taped, and take approximately 60-90 minutes.

Administrative Section

1. TITLE AND SUMMARY OF PROJECT

1.1. Title

1.1.1 What is the formal title of this research proposal?

Evaluation of the relative efficacy and mechanisms of a couples-based intervention for Premenstrual Syndrome through a randomised control trial using mixed methods

1.1.2 What is the short title / acronym of this research proposal (if applicable)?

Evaluating the effectiveness of non-medical treatment for PMS

1.2. Description of the project in plain language

1.2.1 Give a concise and simple description (not more than 400 words), in plain language, of the aims of this project, the proposal research design and the methods to be used to achieve those aims.

The aims of this project are two fold. Firstly, to evaluate the relative efficacy of a brief couple-based PMS intervention, in comparison to a one-to-one PMS intervention, and a self-monitoring control group. The second aim is to identify the effective mechanisms of PMS interventions through process change evaluation. A RCT, and triangulation of methods will be employed to meet the first aim of the study. This allows for the comparison of active treatment with self-monitoring, and the comparison of two active treatments. Process evaluation will also be conducted by the psychologist administering the interventions and the participants to examine mechanisms of treatment effect.

Women will be randomised to one of three conditions, using disproportionate stratified random sampling. The first condition includes a one-to-one PMS intervention that uses a combination of narrative and cognitive-behavioural techniques (Ussher, Hunter et al. 2002). The second condition is a couple-based PMS intervention, developed by the current project team, supported by a UWS internal grant, differs from the above one-to-one PMS intervention primarily in terms of modality (couple versus individual), with the addition of Couples Dialogue techniques (Hendrix 1990. Three monthly 90-minute sessions of therapy and a 2-month booster session will be conducted by a registered clinical psychologist. The third is a control group. Women in this condition will complete daily PMS diaries, as well as evaluation measures at baseline, and 5 months later, to mirror the pre-post evaluation of the treatment conditions.

Outcome measures include the Hospital Anxiety and Depression Scale (Zigmond and Snaith 1983); a daily rating of severity of problems form (Endicott and Harrison 1990); the Dyadic Adjustment Scale (DAS) (Spanier 1976); subjective evaluation of PMS questionnaire (Ussher and Perz 2006); affectional expression subscale of the DAS, clinical observation of couples, and self-report; experience of intervention (will be evaluated post-treatment, using Likert scales and open ended questions.

Semi-structured Narrative Interviews, previously used to examine moderate-severe PMS in both the UK and Australia, will also be employed (Ussher 2003; Perz and Ussher 2006; Ussher, Perz et al. 2007).

Post-intervention, a second interview will be conducted, with the same questions being addressed, with a focus on how the intervention has impacted in each of these areas. Interviews will be audio-taped, and take approximately 60-90 minutes.

2. RESEARCHERS / INVESTIGATORS

2.2. Principal researcher(s) / investigator(s)

2.2.0 How many principal researchers / investigators are there? 3

2.2.1. Principal researcher / investigator 1

2.2.1. Name and contact details

Name: Prof Jane Ussher

Address: University of Western Sydney, Locked Bag 1797
Penrith South DC NSW 1797

Organisation: Gender, Culture and Health: Psyhealth

Area: Psychology

Position: Professor of Women's Health Psychology

Contact: (Bus) 02 9772 6720 (AH) -
(Mob) - (Fax) -

Email: j.ussher@uws.edu.au

2.2.2... Summary of qualifications and relevant expertise NS 4.8.7 NS 4.8.15

BA (Psych) (Hons); PhD; DipClinPsych

Professor Jane Ussher is Professor of Women's Health Psychology, with an international reputation as an authority on gender and health. She has an expertise in a range of methodologies, and has produced groundbreaking work developing theoretical and epistemological frameworks for research in this field that incorporates multiple perspectives, most recently, a critical realist approach. She is also a trained clinical psychologist.

2.2.2... Please declare any general competing interests

None

2.2.2... Name the site(s) for which this principal researcher / investigator is responsible.

University of Western Sydney

2.2.3 Describe the role of the principal researcher / investigator in this project.

Design, data collection, data analysis, and interpretation and dissemination of results

2.2.4 Is the principal researcher / investigator a student? No

2.2.1. Principal researcher / investigator 2

2.2.1. Name and contact details

Name: Dr Janette Perz

Address: University of Western Sydney, Locked Bag 1797
Penrith South DC NSW 1797

Organisation: Gender, Culture and Health: Psyhealth

Area: Psychology

Position: Senior Lecturer

Contact: (Bus) 02 9772 6512 (AH) -
(Mob) - (Fax) -

Email: j.perz@uws.edu.au

2.2.2... Summary of qualifications and relevant expertise NS 4.8.7 NS 4.8.15

BA Hons (Psych), PhD Psychology

Dr Perz is an established researcher with a record of research and scholarly activity demonstrating my commitment to the application of psychology through multi-disciplinary collaborations and approaches. She has a national, and developing international, reputation in the field of Health Psychology, more specifically,

in the areas of reproductive and mental health and early intervention and primary care models. She has initiated, resourced, conducted and supervised research in these key areas of health psychology. Janette has demonstrated expertise in quantitative analyses and have successfully collaborated on an ARC SPIRT and current ARC Discovery and Linkage projects in this capacity. As a health psychology researcher she has made substantial contributions to research projects examining the efficacy of psychological interventions in improving the psychological health and well-being of disadvantaged groups and the detection of depression and anxiety amongst community samples. Dr Perz is an executive board member of several national and state level community mental health associations and have worked closely with community and health groups on research and program evaluation steering and reference committees. Her research activities have been disseminated in refereed academic journals and presented at international and national conferences across the fields of Psychology and Behavioural Medicine. In additon, Dr Perz has supervised to completion 4 PhD, 19 Honours, and 6 professional Masters students with projects in health psychology, and is the Deputy-Director of the Gender, Culture & Health Research Unit: PsyHealth (UWS).

2.2.2... Please declare any general competing interests

None

2.2.2... Name the site(s) for which this principal researcher / investigator is responsible.

University of Western Sydney

2.2.3 Describe the role of the principal researcher / investigator in this project.

Design, data collection, data analysis, and interpretation and dissemination of results

2.2.4 Is the principal researcher / investigator a student? No

2.2.1. Principal researcher / investigator 3

2.2.1. Name and contact details

Name:	Dr Edith Weisberg
Address:	328-336 Liverpool Road<br>Ashfield NSW 2131
Organisation:	Family Planning NSW
Area:	Sydney Centre for Reproductive Health Research
Position:	Director of Research
Contact	(Bus) 02 8752 4342 (AH) -<br>(Mob) - (Fax) -
Email:	edithw@fpahealth.org.au

2.2.2... Summary of qualifications and relevant expertise NS 4.8.7 NS 4.8.15

MBBS(Syd) MM(Syd) FACSHP FRANZCOG

Edith Weisberg is Director of Research at FPA Health, and is a recognised international expert on Women's Health who serves on a nine member International Medical Advisory Panel for the International Planned Parenthood Federation (IPPF), the peak international body on reproductive health with consultative status to the World Health Organisation (WHO). In this capacity she has been involved in providing evidence based advice for women's health issues, including interventions for reproductive distress, contraceptive methods, the prevention of sexually transmitted infections, and adolescent sexuality. Her expertise in Women's Health was recognised by her appointment to an expert working group of WHO to develop evidence based practice guidelines for health professionals worldwide. She has contributed widely to increasing knowledge amongst health professionals especially GPs in the area of reproductive health and sexuality by lecturing in post-graduate courses at FPA Health and the University of Sydney. She has designed and carried out a number of surveys on GP attitudes and practice in fertility control. She has had a long-standing interest in problems associated with the menstrual cycle and is at present involved in an international study on management of dysfunctional uterine bleeding. She has been involved in mixed method research, including an ARC SRIRT grant on men's sexuality and a Dept Health funded project on cervical screening, both with CI1 (Ussher). She has also participated in studies of premenstrual syndrome, including both medical and self-help interventions (the latter with CI1), and has had extensive experience in managing PMS while in private practice. She will provide expertise on clinical management of PMS, and facilitate recruitment through FPA Health, in the current study.

2.2.2... Please declare any general competing interests

None

2.2.2... Name the site(s) for which this principal researcher / investigator is responsible.

Family Planning NSW

2.2.3 Describe the role of the principal researcher / investigator in this project.
Design, data collection, data analysis, and interpretation and dissemination of results

2.2.4 Is the principal researcher / investigator a student? No

2.3. Associate researcher(s) / investigator(s)

2.3.1 How many known associate researchers are there? (You will be asked to give contact details for these associate researchers / investigators at question 2.3.1.1) 1

2.3.2 Do you intend to employ other associate researchers / investigators? No

2.3.1...Associate Researchers / Investigators 1

2.3.1...Name and contact details

Name:	Dr Yasmin Hawkins
Address:	University of Western Sydney, Locked Bag 1797<br>Penrith South DC NSW 1797
Organisation:	Gender, Culture, and Health: PsyHealth
Area:	School of Psychology
Position:	Senior Research Project Officer
Contact	(Bus) + 61 2 9772 6476 (AH) -<br>(Mob) - (Fax) +61 2 9772 6757
Email:	y.hawkins@uws.edu.au

2.3.1... Summary of qualifications and relevant expertise NS 4.8.7 NS 4.8.15
BA Hons (Psych): PhD Psychology
Dr Yasmin Hawkins is a Research Project Officer for the Gender, Culture &
Health Research: PsyHealth. Yasmin has substantial experience in the technique of interviewing, data collection and analysis. She has training in a range of qualitative research designs, and in the analysis of qualitative research data.

2.3.1... Please declare any general competing interests
None

2.3.1... Description of the role of the associate researcher / investigator in this project.
Assitance with data collection, data analysis, and interpretation and dissemination of results.

2.3.1... Name the site at which the associate researcher / investigator has responsibility.
The University of Western Sydney

2.3.1... Is this associate researcher / investigator a student? No

2.4. Contact

Provide the following information for the person making this application to the HREC.

2.4.1. Name and contact details

Name:	Dr Janette Perz
Address:	University of Western Sydney, Locked Bag 1797<br>Penrith South DC NSW 1797
Organisation:	Gender, Culture and Health: Psyhealth
Area:	Psychology
Position:	Senior Lecturer
Contact	(Bus) 02 9772 6512 (AH) -<br>(Mob) - (Fax) -
Email:	j.perz@uws.edu.au

2.5. Other personnel relevant to the research project

2.5.1 How many known other people will play a specified role in the conduct of this research project? 1

2.5.1... Describe the role, and expertise where relevant (e.g. counsellor), of these other personnel.
PhD Candidate Marlee King will assist with data collection activities, analysis and dissemination.

2.5.2 Is it intended that other people, not yet known, will play a specified role in the conduct of this research project? No

2.6. Certification of researchers / investigators

2.6.1 Are there any relevant certification, accreditation or credentialing requirements relevant to the conduct of this research? No

2.7. Training of researchers / investigators

2.7.1 Do the researchers / investigators or others involved in any aspect of this research project require any additional training in order to undertake this research? No

3. RESOURCES

3.1. Project Funding / Support

3.1.1. Indicate how the project will be funded

3.1.1... Type of funding.
[Please note that all fields in any selected funding detail column (with the exception of the code) will need to be completed.]

	**External Competitive Grant**
Name of Grant / Sponsor	ARC Discovery Project
Amount of funding	$420, 000
Confirmed / Sought	Confirmed
Detail in kind support	FPA Health will assist with recruitment and provide clinical space for interviews.
Indicate the extent to which the scope of this HREC application and grant are aligned	The HREC and ARC application are fully aligned

3.1.1... How will you manage a funding shortfall (if any)?
There is sufficient funding for the proposal from the ARC discovery project grant.

3.1.2 Will the project be supported in other ways eg. in-kind support/equipment by an external party eg. sponsor Yes

3.1.2... Describe the support and indicate the provider.
FPA Health will assist with recruitment and provide clinical space for interviews.

3.2. Duality of Interest

3.2.1 Describe any commercialisation or intellectual property implications of the funding/support arrangement.
N/A

3.2.2 Does the funding/support provider(s) have a financial interest in the outcome of the research? No

3.2.3 Does any member of the research team have any affiliation with the provider(s) of funding/support, or a financial interest in the outcome of the research? No

3.2.4 Does any other individual or organisation have an interest in the outcome of this research No

3.2.5 Are there any restrictions on the publication of results from this research? No

4. PRIOR REVIEWS

4.1. Ethical review

4.1.0. Duration and location

4.1.0... In how many Australian sites, or site types, will the research be conducted? 1

4.1.0... In how many overseas sites, or site types, will the research be conducted? 0

Provide the following information for each site or site type (Australian and overseas, if applicable) at which the research is to be conducted

4.1.0...Site / Site Type 1

4.1.0... Site / Site Type Name
The University of Western Sydney

4.1.0... Site / Site Type Location
Bankstown Campus, Milperra, 2214, NSW

4.1.0...Provide the start and finish dates for the whole of the study including data analysis

Anticipated start date 01/01/2009

Anticipated finish date 31/12/2012

4.1.0... Are there any time-critical aspects of the research project of which an HREC should be aware? No

4.1.1 To how many Australian HRECs (representing site organisations or the researcher's / investigator's organisation) is it intended that this research proposal be submitted? 1

4.1.1...HREC 1

4.1.1... Name of HREC University of Western Sydney Human Research Ethics Committee (EC00314)

4.1.1...Provide the start and finish dates for the research for which this HREC is providing ethical review.

Anticipated start date or date range 01/12/2008

Anticipated finish date or date range 31/12/2012

4.1.1... For how many sites at which the research is to be conducted will this HREC provide ethical review? 1

4.1.1...Site 1

4.1.1... Name of site The University of Western Sydney

4.1.1... Which of the researchers / investigators involved in this project will conduct the research at this site?

**Principal Researcher(s)**	**Associate Researcher(s)**
Prof Jane Ussher	Dr Yasmin Hawkins
Dr Janette Perz	

4.1.2 Have you previously submitted an application, whether in NEAF of otherwise, for ethical review of this research project to any other HRECs? No

4.3. Peer review

4.3.1 Has the research proposal, including design, methodology and evaluation undergone, or will it undergo, a peer review process? NS 1.2 Yes

4.3.1... Provide details of the review and the outcome. A copy of the letter / notification, where available, should be attached to this application.
The research is fully funded by an ARC Discovery Grant and underwent peer review in the assessment and determination of the submission.

Ethical Review Section

Summary

Applicant / Principal Researcher(s)

Dr Janette Perz

BA Hons (Psych), PhD Psychology
Dr Perz is an established researcher with a record of research and scholarly activity demonstrating my commitment to the application of psychology through multi-disciplinary collaborations and approaches. She has a national, and developing international, reputation in the field of Health Psychology, more specifically, in the areas of reproductive and mental health and early intervention and primary care models. She has initiated, resourced, conducted and supervised research in these key areas of health psychology. Janette has demonstrated expertise in quantitative analyses and have successfully collaborated on an ARC SPIRT and current ARC Discovery and Linkage projects in this capacity. As a health psychology researcher she has made substantial contributions to research projects examining the efficacy of psychological interventions in improving the psychological health and well-being of disadvantaged groups and the detection of depression and anxiety amongst community samples. Dr Perz is an executive board member of several national and state level community mental health associations and have worked closely with community and health groups on research and program evaluation steering and reference committees. Her research activities have been disseminated in refereed academic journals and presented at international and national conferences across the fields of Psychology and Behavioural Medicine. In additon, Dr Perz has supervised to completion 4 PhD, 19 Honours, and 6 professional Masters students with projects in health psychology, and is the Deputy-Director of the Gender, Culture & Health Research Unit: PsyHealth (UWS).

Potential conflicts of interest
None

Prof Jane Ussher

BA (Psych) (Hons); PhD; DipClinPsych

Professor Jane Ussher is Professor of Women's Health Psychology, with an international reputation as an authority on gender and health. She has an expertise in a range of methodologies, and has produced groundbreaking work developing theoretical and epistemological frameworks for research in this field that incorporates multiple perspectives, most recently, a critical realist approach. She is also a trained clinical psychologist.

Potential conflicts of interest
None

Dr Edith Weisberg

MBBS(Syd) MM(Syd) FACSHP FRANZCOG
Edith Weisberg is Director of Research at FPA Health, and is a recognised international expert on Women's Health who serves on a nine member International Medical Advisory Panel for the International Planned Parenthood Federation (IPPF), the peak international body on reproductive health with consultative status to the World Health Organisation (WHO). In this capacity she has been involved in providing evidence based advice for women's health issues, including interventions for reproductive distress, contraceptive methods, the prevention of sexually transmitted infections, and adolescent sexuality. Her expertise in Women's Health was recognised by her appointment to an expert working group of WHO to develop evidence based practice guidelines for health professionals worldwide. She has contributed widely to increasing knowledge amongst health professionals especially GPs in the area of reproductive health and sexuality by lecturing in post- graduate courses at FPA Health and the University of Sydney. She has designed and carried out a number of surveys on GP attitudes and practice in fertility control. She has had a long-standing interest in problems associated with the menstrual cycle and is at present involved in an international study on management of dysfunctional uterine bleeding. She has been involved in mixed method research, including an ARC SRIRT grant on men's sexuality and a Dept Health funded project on cervical screening, both with CI1 (Ussher). She has also participated in studies of premenstrual syndrome, including both medical and self-help interventions (the latter with CI1), and has had extensive experience in managing PMS while in private practice. She will provide expertise on clinical management of PMS, and facilitate recruitment through FPA Health, in the current study.

Potential conflicts of interest
None

Associate Researcher(s) / Investigator(s)

Dr Yasmin Hawkins

BA Hons (Psych): PhD Psychology
Dr Yasmin Hawkins is a Research Project Officer for the Gender, Culture & Health Research: PsyHealth. Yasmin has substantial experience in the technique of interviewing, data collection and analysis. She has training in a range of qualitative research designs, and in the analysis of qualitative research data.

Potential conflicts of interest
None

5. PROJECT

5.1. Type of Research

5.1.1 Tick as many of the following 'types of research' as apply to this project. Your answers will assist HRECs in considering your proposal. A tick in some of these boxes will generate additional questions relevant to your proposal (mainly because the National Statement requires additional ethical matters to be considered), which will appear in Section 9 of NEAF.

This project involves:

[X] Research using qualitative methods NS 3.1

[X] Research using quantitative methods, population level data or databanks, e.g survey research, epidemiological research NS 3.2

5.1.2 Does the research involve limited disclosure to participants? NS 2.3 — No

5.1.3 Are the applicants asking the HREC / review body to waive the requirement of consent? NS 2.3.5 — No

5.2. Research plan

5.2.1 Describe the theoretical, empirical and/or conceptual basis, and background evidence, for the research proposal, eg. previous studies, anecdotal evidence, review of literature, prior observation, laboratory or animal studies (4000 character limit). NS 1.1

Premenstrual distress is now widely recognised to be a major social and health problem, with epidemiological surveys estimating that up to 40% of women experience moderate distress, categorised by clinicians and researchers as premenstrual syndrome (PMS), and 11-13% experience severe distress and disruption to their lives, categorised as premenstrual dysphoric disorder (PMDD) (Steiner & Born, 2000). Recent research by members of the project team has demonstrated that PMS is an inter-subjective phenomenon, a continuum of experience that develops and is constructed within significant family relationships (Ussher, 2002b, 2003b). A small-scale pilot study has recently been completed by members of the project team, to focus specifically on the association between PMS and relationships, in couples attending Relationships Australia for relationship counselling and those recruited from a more general population (Ussher, Perz, & James, forthcoming). Through narrative interviews, women reported difficulties in relationships being exacerbated premenstrually, with both partners positioning ‘PMS’ as to blame. The association between self-policing (Jack, 1991), adult attachment style (Feeney, 1990), and PMS, was also examined, with high levels of self-silencing, and insecure attachment styles found in women who positioned themselves as ‘PMS sufferers’, compared to population norms. Women’s partner’s perspectives were also examined in a small sub-sample, demonstrating that few men knew how to respond to premenstrual anger or depression. The current project will extend and develop this pilot research, examining the development, experience, and construction of PMS within a broader population of women and their partners, across different relationship types and contexts.

5.2.2 State the aims of the research and the research question and/or hypotheses, where appropriate.

The aims of this project are to draw on and augment an ongoing program of PMS research through:
1. Evaluating the relative efficacy of a brief couple-based PMS intervention, in comparison to a one-to-one PMS intervention, and a self-monitoring control group, within a RCT, using the triangulation of qualitative and quantitative methods.
2. Identifying the effective mechanisms of PMS interventions through process change evaluation.
Hypotheses: When compared to individual therapy, couple therapy will lead to greater reduction in relational issues associated with PMS, more effective coping, lower premenstrual distress, lower depression and anxiety, and greater relationship satisfaction. Both interventions will be more effective than the control condition, on the above variables.

5.2.3 Has this project been undertaken previously? — No

5.3. Benefits/Risks

5.3.0 Does the research involve a practice or intervention which is an alternative to a standard practice or intervention? — No

5.3.2 What expected benefits (if any) will this research have for the wider community?

The value of this project is in providing a greater understanding of the impact of premenstrual symptomatology on relationships, an area of research which has not, as yet, been systematically explored in an in-depth qualitative manner. This will lead to new insights about the development and course of PMS, thus providing important social benefits; theoretical and methodological advances that could be applied to other areas of women’s mental and physical health; and, the development of university/community partnerships. This project will provide a framework for the development of programs of prevention and

intervention that include partners and other family members, which can be utilised by both Family Planning NSW, and by other agencies. The findings of this project will be disseminated through the publication of academic and clinical papers and through presentations at relevant national and international conferences. They will also be disseminated, in a plain English summary, through Women's Health Organisations throughout Australia and through targeted media outlets. The people who stand to benefit from this project are women with premenstrual symptomatology, couples where a woman has premenstrual symptomatology, and couples counsellors and psychologists who will be informed by the results of the research.

5.3.3 What expected benefits (if any) will this research have for participants? NS 2.1

The value of this project is in providing a greater understanding of the impact of premenstrual symptomatology on relationships, an area of research which has not, as yet, been systematically explored in an in-depth qualitative manner. This will lead to new insights about the development and course of PMS, thus providing important social benefits; as well as theoretical and methodological advances that could be applied to other areas of women's mental and physical health.

The women who participant in this research are all currently experiencing distress around their premenstrual change. These women stand to benefit from the broader findings of the project, either through the development of self help resources for women and couples, through couples counsellors and psychologists who will be informed by the results of the research, or through future research that builds on the findings of this project. In addition, women and their partners may benefit through having an opportunity to discuss issues that are often not discussed, and to gain support through the knowledge that others have had similar experiences. After taking part in the research, all participants will be a given a copy of a self-help pack for PMS, developed by one of the CIs, and evaluated in a previous project conducted in partnership with FPA Health, and funded by a UWS Partnership grant.

5.3.4 Are there any risks to participants as a result of participation in this research project? NS 2.1 — Yes

5.3.5 Explain how the likely benefit of the research justifies the risks of harm or discomfort to participants. NS 1.6

Whilst no psychological stress or distress is expected, and the intervention sessions and interviews will be conducted by trained and experienced psychologists, it is possible that participants may raise issues during the sessions that they would like to explore further in a therapeutic setting. The researchers will refer participants on to agencies, such as Family Planning NSW and Relationships Australia, who can deal with such issues, if this is requested or seems appropriate.

If participants exhibit discomfort or distress during interventions sessions or interviews, they will be reminded that they can take a break or stop completely if they wish, or that the topic under discussion can be dropped and the session continued on a different topic.

Participants will have the opportunity to express any positive or negative aspects of the project, and will be explicitly reminded of their right to withdraw from the study at any time.

5.3.8 Are there any other risks involved in this research? eg. to the research team, the organisation, others — No

5.3.9 Is it anticipated that the research will lead to commercial benefit for the investigator(s) and or the research sponsor(s)? — No

5.3.11 Is there a risk that the dissemination of results could cause harm of any kind to individual participants - whether their physical, psychological, spiritual, emotional, social or financial well-being, or to their employability or professional relationships - or to their communities? — No

5.4. Monitoring

Refer to NS 3.3.19 - 3.3.25

5.4.1 What mechanisms do the researchers / investigators intend to implement to monitor the conduct and progress of the research project? NS 5.5

The principle researchers will meet regularly to ensure the optimal conduct and progress of the research project. Annual progress reports will be submitted to the ARC.

6. PARTICIPANTS

6.1. Research participants

6.1.1 The National Statement identifies the need to pay additional attention to ethical issues associated with research involving certain specific populations.

This question aims to assist you and the HREC to identify and address ethical issues that are likely to arise in your research, if its design will include one or more of these populations. Further, the National Statement recognizes the cultural diversity of Australia's population and the importance of respect for that diversity in the recruitment and involvement of participants. Your answer to this question will guide you to additional questions (if any) relevant to the participants in your study.

6.1.1 Tick as many of the following 'types of research participants' who will be included because of the project design, or their inclusion is probable, given the diversity of Australia's population. If none apply, please indicate this below.

	c) Design specifically excludes
Children and/or young people (ie. <18 years)<br>NS 4.2	[X]

6.2. Participant description

6.2.1 How many participant groups are involved in this research project? 5

6.2.2 What is the expected total number of participants in this project at all sites?
250

6.2.3. Group 1

6.2.3... Group name for participants in this group
Screening Group

6.2.3... Expected number of participants in this group
130

6.2.3... Age range
18-48

6.2.3... Other relevant characteristics of this participant group
Women who self-identify as experiencing PMS but not to a moderate-severe level. These participants will complete a pre-screening questionnaire only and not proceed to further stages in the research.

6.2.3... Why are these characteristics relevant to the aims of the project?
Treatment study for PMS

6.2.3. Group 2

6.2.3... Group name for participants in this group
Individual PMS intervention

6.2.3... Expected number of participants in this group
40

6.2.3... Age range
18-48

6.2.3... Other relevant characteristics of this participant group
Women who self-identify as experiencing moderate to severe PMS, whose partners are also prepared to take part in an intervention.

6.2.3... Why are these characteristics relevant to the aims of the project?
Treatment study for PMS

6.2.3. Group 3

6.2.3... Group name for participants in this group
Couple-based PMS intervention

6.2.3... Expected number of participants in this group
40

6.2.3... Age range
18-48

6.2.3... Other relevant characteristics of this participant group

Women who self-identify as experiencing moderate to severe PMS, whose partners are also prepared to take part in an intervention.

6.2.3... Why are these characteristics relevant to the aims of the project?
Treatment study for PMS

6.2.3. Group 4

6.2.3... Group name for participants in this group
Wait-List Control group

6.2.3... Expected number of participants in this group
40

6.2.3... Age range
18-48

6.2.3... Other relevant characteristics of this participant group
Women who self-identify as experiencing moderate to severe PMS, whose partners are also prepared to take part in an intervention.

6.2.3... Why are these characteristics relevant to the aims of the project?
Treatment study for PMS.

6.2.3. Group 5

6.2.3... Group name for participants in this group
Interview participants

6.2.3... Expected number of participants in this group
30

6.2.3... Age range
18-48

6.2.3... Other relevant characteristics of this participant group
Drawn from participants in the 3 intervention groups.

6.2.3... Why are these characteristics relevant to the aims of the project?
To assess the efficacy of the interventions.

6.2.4. Your response to questions at Section 6.1 - Research Participants indicates that the following participant groups are excluded from your research. If this is not correct please return to section 6.1 to amend your answer.

Children and/or young people (ie. <18 years)

6.2.4... Have any particular potential participants or groups of participants been excluded from this research? In answering this question you need to consider if it would be unjust to exclude these potential participants. NS 1.4
The inclusion criteria will be: self-identified age between 18 and 48 years; having regular menstrual cycles (21-35 days); presently not taking hormonal or psychotropic medication, or having been diagnosed with a major psychiatric illness (Axis I and II); not being pregnant or lactating within the previous 12 months; and the self-reported presence of moderate-severe premenstrual symptoms. Women who do not meet these criteria will be excluded. This is not unjust as the targeted intervention requires efficacy testing on a targeted clinical population.
As a consequence of these inclusion criteria, children and people <18 years have been excluded.

6.3. Participation experience

6.3.1 Provide a concise detailed description, in not more than 200 words, in terms which are easily understood by the lay reader of what the participation will involve.
Participants will be randomly allocated to one of the following groups:
Couple-based intervention:
The woman and her partner will meet with a trained psychologist once a month for 3 months, and also for a 2 month follow-up, for approximately 90 minutes each time (4 meetings total). They will discuss the woman's PMS, and strategies of coping, as well as ways in which the partner can support help the woman to reduce her premenstrual distress.
One-to-one intervention:
This intervention involves the same strategies, but is conducted one-to-one with the woman who experiences PMS. She will meet with a trained psychologist once a month for 3 months, and also for a 2 month follow-up, for approximately 90 minutes each time (4 meetings total). She will discuss her PMS, and strategies of coping, as well as ways in which her partner can support her and help to reduce her

premenstrual distress.
Wait-list control group:
The third group will be given a self-help pack with the additional option of attending a group meeting with a psychologist to discuss strategies of premenstrual coping. The self-help pack involves information about the causes of PMS plus strategies to help cope.

All women who take part will be asked to complete a brief daily moods diary for the 6 month duration of the study. Additionally, during the course of the study, women will be asked to complete a questionnaire about their premenstrual experiences at three different times. They will be given instructions on how to complete the questionnaires and a reply-paid envelope to return them or given the option to complete the questionnaire online.

A sub-set of participants will take part in 3 semi-structured face-to-face interviews with a researcher (not the person who delivered the intervention) focusing on the experience and construction of PMS. Interviews will be audio-taped, and take approximately 60-90 minutes.

6.4. Relationship of researchers / investigators to participants

6.4.1 Specify the nature of any existing relationship or one likely to rise during the research, between the potential participants and any member of the research team or an organisation involved in the research.

There are no relationships between the potential participants and the researchers. Recruitment will be conducted in locations where some participants in these groups may be in the receipt of current or previous professional care (eg. Family Planning NSW). This notwithstanding, there is no association between this care and the conduct of this research and thus there will be no compromise to their care from the service.

6.4.2 Describe what steps, if any, will be taken to ensure that the relationship does not impair participants' free and voluntary consent and participation in the project.

If a relationship between participant and researcher is identified, the participant will have the right to withdraw their involvement from the project without penalty or prejudice.

6.4.3 Describe what steps, if any, will be taken to ensure that decisions about participation in the research do not impair any existing or foreseeable future relationship between participants and researcher / investigator or organisations.

The rights of participants will be protected via informed consent procedures.

6.4.4 Will the research impact upon, or change, an existing relationship between participants and researcher / investigator or organisations.? No

6.4.5 Is it intended that the interview transcript will be shown or made available to participants? 3.1.15 No

6.5. Recruitment

6.5.1 What processes will be used to identify potential participants?

Participants will be recruited from a range of contexts: a) Participants in a current ARC project (HREC 05/125) who indicated on further contact forms that they are interested in participating in a treatment study. The study has a data base of 192 women with PMS, 50% of whom have indicated such an interest; b) Clients at Family Planning NSW, GP surgeries, and Women’s Health Centres in NSW; c) Advertisements in local press, relevant online sites, and women’s health publications.

6.5.2 Is it proposed to 'screen' or assess the suitability of the potential participants for the study? Yes

6.5.2... How will this be done?

In response to a written invitation, potential participants asked to complete a brief self-report questionnaire about their premenstrual experiences to determine the presence of moderate-severe premenstrual symptoms. If a woman's premenstrual experiences are mild, they will be given information about a self-help PMS package and not included in any further stage of this study. If their premenstrual changes are moderate-severe, they will be randomly allocated to one of study groups.

6.5.3 Describe how initial contact will be made with potential participants.

A member of the research team will contact a potential participant where the participant has initiated contact by responding to a written or published invitation to participation as detailed in an information sheet or advertisement for the study.

6.5.3... Do you intend to include both males and females in this study? Yes

6.5.3... What is the expected ratio of males to females that will be recruited into this study and does this ratio accurately reflect the distribution of the disease, issue or condition within the general community?

For every woman who is recruited for the study, her partner, male or female, will also be invited to take part in the intervention. 40 dyads will be allocated to the couple-based intervention group. Whilst partners (many of whom will be men) will be involved as participants in the couple-based intervention, data will not be

collected from them. With the focus of the study on PMS experiences, outcome data will be collected from females only.

6.5.4 Is an advertisement, e-mail, website, letter or telephone call proposed as the form of initial contact with potential participants? Yes

6.5.4... Provide details and a copy of text/script.

See attachment.

6.5.5 If it became known that a person was recruited to, participated in, or was excluded from the research, would that knowledge expose the person to any disadvantage or risk? No

6.6. Consent process

6.6.1 Will consent for participation in this research be sought from all participants? Yes

6.6.1... Will there be participants who have capacity to give consent for themselves? Yes

6.6.1... What mechanisms/assessments/tools are to be used, if any, to determine each of these participant's capacity to decide whether or not to participate?

Each potential participant will be given the information sheet to read to determine if they would like to participate in the research. Self-assessment by the participant will be used to determine capacity to consent, following a reading of the information sheet.

6.6.1... Are any of the participants children or young people? No

6.6.1... Will there be participants who do not have capacity to give consent for themselves? No

6.6.1... Describe the consent process, ie how participants or those deciding for them will be informed about, and choose whether or not to participate in, the project.

Participants will be provided with information about the aims of the project and how the data they provide will be treated by the principal researchers. Prior to their participation in the research, the participants will be given an information sheet and informed consent form to sign - outlining their ethical rights, the nature of the research, contact details for the principal researchers should they wish to seek further information, discuss the research or withdraw their participation.

6.6.1... If a participant or person on behalf of a participant chooses not to participate, are there specific consequences of which they should be made aware, prior to making this decision? 4.6.6 - 4.6.7

No

6.6.1... Might individual participants be identifiable by other members of their group, and if so could this identification expose them to risks?

No

6.6.1... If a participant or person on behalf of a participant chooses to withdraw from the research, are there specific consequences of which they should be made aware, prior to giving consent?

No

6.6.1... Specify the nature and value of any proposed incentive/payment (eg. movie tickets, food vouchers) or reimbursement (eg travel expenses) to participants.

Group 5 participants will be offered 3 x $30 reimbursement to cover the costs associated with reasonable reimbursement for travel to attend pre, post and follow-up interviews.

6.6.1... Explain why this offer will not impair the voluntary nature of the consent, whether by participants' or persons deciding for their behalf. NS 2.2.10 - 2.2.11

Information sheets will be distributed to participants prior to their travel to be involved in the research process. Thus, the incentive will not impair the voluntary nature of consent. The offer of $30 is to cover costs associated with travel, and is a reasonable reimbursement for their travel costs rather than an incentive to participate.

6.6.3 Do you propose to obtain consent from individual participants for your use of their stored data/samples for this research project? No

6.6.3... Give justification

No stored data/samples from participants are sought.

8. CONFIDENTIALITY/PRIVACY

8.1. Do privacy guidelines need to be applied in the ethical review of this proposal?

8.1.1 Indicate whether the source of the information about participants which will be used in this research project will involve:

[X] collection directly from the participant

8.1.1... Information which will be collected for this research project directly from the participant

8.1.1... Describe the information that will be collected directly from participants. Be specific where appropriate.

The data that will be collected from participants includes questionnaires examining psychological well-being, premenstrual symptoms, and relationship satisfaction. Specially, outcome measures include the Hospital Anxiety and Depression Scale (Zigmond and Snaith 1983); Daily rating of severity of problems form (Endicott and Harrison 1990); the Dyadic Adjustment Scale (DAS) (Spanier 1976); Subjective evaluation of PMS questionnaire (Ussher and Perz 2006);Silencing the Self Scale (STSS) (Jack, 1991).Couple Communication is assessed through the affectional expression subscale of the DAS, clinical observation of couples, and self-report; experience of intervention (for women in the active treatment conditions) will be evaluated post-treatment, using Likert scales and open ended questions.

Interview data will focus on the experience and construction of PMS, as well as any changes in the relationship. Women are asked to describe the course and development of premenstrual distress and recount a typical experience of 'PMS' in the context of relationships. Post-intervention, a followup interviews will be conducted, with similiar questions being addressed, with a focus on how the intervention has impacted in each of these areas.

8.1.1... The information collected by the research team about participants will be in the following form(s). Tick more than one box if applicable.

[X] re-identifiable

8.1.1... Give reasons why it is necessary to collect information in individually identifiable or re-identifiable form.

The research design is carefully structured so that each stage generates a heuristic framework to inform the inquiry techniques in following stages. Consequently, participants who take part in screening stage of the research will be asked about their willingness to take part in intervention stage the research. In order to recontact participants for the intervention, identifiable information about participants (their contact details) will be stored in coded form on computer file accessible only by the researchers. Additionally, the pre, post and follow-up assessments require that matched data is used. For this purpose, data will be coded to allow for analysis.

8.1.1... Consent process

You have indicated that you will be varying the conditions of or waiving consent. See questions in section 6.6

8.1.1... Will consent be specific or extended or unspecified? NS 2.2.14 - 2.2.18 Specific

8.1.1... Provide reasons why this form of consent has been chosen. You may need to revise your answer at 6.6.1.1.3 to provide details on the consent process

Participants are asked to consent to involvement in the current project. Expressions of interest will be sought from participants who may choose to become in further research for which specific consent will be sought.

8.2. Using information from participants

8.2.1 Describe how information collected about participants will be used in this project.

All information collected about participants will be de-identified when data entered, analyzed and reported. For quantitative information, data will will be aggregated and qualitative data will be assessed for themes and patterns. Analyzed data will be disseminated through research publications and conference presentations.

8.2.2 Will any of the information used by the research team be in identified or re-identifiable (coded) form? Yes

8.2.2... Indicate whichever of the following applies to this project:

[X] Information collected for, used in, or generated by, this project is intended to be used for establishing a database/data collection/register for future use by the researcher for which ethical approval will be sought.
[X] Information collected for, used in, or generated by, this project will/may be made available to a third

party for a subsequent use for which ethical approval will be sought.

8.2.4 List ALL research personnel and others who, for the purposes of this research, will have authority to use or have access to the information and describe the nature of the use or access. Examples of others are: student supervisors, research monitors, pharmaceutical company monitors .

The PIs, AI and PhD candidate will be the only people with access to the data. De-identified data will be accessed for qualitative and quantitative analyzes.

8.3. Storage of information about participants during and after completion of the project

8.3.1 In what formats will the information be stored during and after the research project? (eg. paper copy, computer file on floppy disk or CD, audio tape, videotape, film)

Audio tapes, hard copy questionnaires and interview transcripts, electronic text and audio files.

8.3.2 Specify the measures to be taken to ensure the security of information from misuse, loss, or unauthorised access while stored during and after the research project? (eg. will identifiers be removed and at what stage? Will the information be physically stored in a locked cabinet?)

All information provided by participants will be treated with respect, and confidentiality will be maintained. Only the named investigators will have access to participant interviews and data. All interviews and questionnaires will be de-identified, and numeric codes or pseudonyms used in the analysis. Participants will not be identified in the publication of any material or findings related to the research; pseudonyms will be utilised in the reporting of qualitative data. The tapes and paper based data will be stored securely in locked filing cabinets, and electronic files stored on computers.

8.3.5 The information which will be stored at the completion of this project is of the following type(s). Tick more than one box if applicable.

[X] non-identifiable

8.3.6 For how long will the information be stored after the completion of the project and why has this period been chosen?

All materials will be kept for a minimum of five years after the completion of the project or publication.

8.3.7 What arrangements are in place with regard to the storage of the information collected for, used in, or generated by this project in the event that the principal researcher / investigator ceases to be engaged at the current organisation?

In the event that one of the principal researchers ceases to be engaged at the University of Western Sydney, the data will remain with the other PIs at the University as per the storage procedures outlined in earlier responses.

8.4. Ownership of the information collected during the research project and resulting from the research project

8.4.2 Who is understood to own the information resulting from the research, eg. the final report or published form of the results?

The Principal Researchers and UWS own the information resulting from the research project. The PhD candidate will own the copyright to their thesis.

8.4.3 Does the owner of the information or any other party have any right to impose limitations or conditions on the publication of the results of this project? No

8.5. Disposal of the information

8.5.1 Will the information collected for, used in, or generated by this project be disposed of at some stage? Yes

8.5.1... At what stage will the information be disposed?

All materials will be kept for a minimum of five years after the completion of the project or publication.

8.5.1... How will information, in all forms, be disposed?

All materials will be securely disposed of by shredding questionnaires and erasing tapes before destruction.

8.6. Reporting individual results to participants and others

8.6.1 Is it intended that results of the research that relate to a specific participant be reported to that participant? No

8.6.1... Explain/justify why results will not be reported to participants.

The data that informs this project will be aggregated across participants. Therefore there will be no results reported that relate to a specific participant.

8.6.2 Is the research likely to produce information of personal significance to individual participants? No

8.6.3 Will individual participant's results be recorded with their personal records? No

8.6.4 Is it intended that results that relate to a specific participant be reported to anyone other than that participant? No

8.6.5 Is the research likely to reveal a significant risk to the health or well being of persons other than the participant, eg family members, colleagues No

8.6.6 Is there a risk that the dissemination of results could cause harm of any kind to individual participants - whether their physical, psychological, spiritual, emotional, social or financial well-being, or to their employability or professional relationships - or to their communities? No

8.6.7 How is it intended to disseminate the results of the research? eg report, publication, thesis

1. Annual progress report to ARC with a final report at the end of the project and an Executive Summary for public dissemination. 2. Presentation of the research findings through a series of refereed articles in academic journals and at national and international conferences and a possible monograph 3. Dissemination of main findings through the Websites associated with the research team. 5. The findings will be released through targeted media outlets to maximise their impact of the findings. De-identified data will be used in all disseminated outputs.

8.6.8 Will the confidentiality of participants and their data be protected in the dissemination of research results? Yes

8.6.8... Explain how confidentiality of participants and their data will be protected in the dissemination of research results

All information collected about participants will be de-identified when data entered, analyzed and reported. For quantitative information, data will will be aggregated and qualitative data will be assessed for themes and patterns. Analyzed data will be disseminated through research publications and conference presentations.

9. DECLARATIONS AND SIGNATURES

9.1 Project Title

Evaluation of the relative efficacy and mechanisms of a couples-based intervention for Premenstrual Syndrome through a randomised control trial using mixed methods

9.2 Human Research Ethics Committee to which this application is made

University of Western Sydney Human Research Ethics Committee (EC00314)

9.3 Signatures and undertakings

Applicant / Principal Researchers (including students where permitted)

I/we certify that:

- All information is truthful and as complete as possible.
- I/we have had access to and read the National Statement on Ethical Conduct in Research Involving Humans.
- the research will be conducted in accordance with the National Statement.
- the research will be conducted in accordance with the ethical and research arrangements of the organisations involved.
- I/we have consulted any relevant legislation and regulations, and the research will be conducted in accordance with these.
- I/we will immediately report to the HREC anything which might warrant review of the ethical approval of the proposal NS 5.5.3 including:
  - serious or unexpected adverse effects on participants;
  - proposed changes in the protocol; and
  - unforseen events that might affect continued ethical acceptability of the project.
- I/we will inform the HREC, giving reasons, if the research project is discontinued before the expected date of completion NS 5.5.6 see NS 5.5.8(b);
- I/we will adhere to the conditions of approval stipulated by the HREC and will cooperate with HREC monitoring requirements. At a minimum annual progress reports and a final report will be provided to the HREC.

Applicant / Chief Researcher(s) / Principal Researcher(s)

Dr Janette Perz<br>Gender, Culture and Health: Psyhealth	______________________<br>Signature	____/____/______<br>Date
Prof Jane Ussher<br>Gender, Culture and Health: Psyhealth	______________________<br>Signature	____/____/______<br>Date
Dr Edith Weisberg<br>Family Planning NSW	______________________<br>Signature	____/____/______<br>Date

Associate Researchers

Dr Yasmin Hawkins	______________________<br>Signature	____/____/______<br>Date

Heads of departments/schools/research organisation

I/we certify that:

- I/we are familiar with this project and endorse its undertaking;
- the resources required to undertake this project are available;
- the researchers have the skill and expertise to undertake this project appropriately or will undergo appropriate training as specified in this application.

__________ ____________________ ____________________________________
Title First name Surname

____________________________________ ____________________________________
Position Organisation name

____/____/____ ____________________________________
Date Signature

10. ATTACHMENTS

This page and all pages that follow don't need to be submitted to your HREC.

10.1 List of Attachments

Core Attachments	Attachments which may be required/appropriate.
Recruitment/invitation	Copy of advertisement, letter of invitation etc
Participant Information	Copy or script for participant<br>Copy or script for parent, legal guardian or person responsible as appropriate
Consent Form	Copy for participant<br>For parent, legal guardian or person responsible as appropriate<br>For, optional components of the project eg. genetic sub study
Peer review	Copy of peer review report or grant submission outcome
HREC approvals	Copy of outcome of other HREC reviews

Attachments specific to project or participant group	Attachments which may be required/appropriate.
Research conducted in the workplace or possibly impacting on workplace relationships	Evidence of support/permission from workplace where research will be conducted
Epidemiological research	Evidence of support/permission from database custodian for proposed access / use of data
Children and/or young people (ie. <18 years)	Information/consent form for parent, legal guardian or person responsible

10.2 Participant information elements
Core Elements
Provision of information to participants about the following topics should be considered for all research projects.

Core Elements	Issues to consider in participant information
About the project	Full title and / or short title of the project<br>Plain language description of the project<br>Purpose / aim of the project and research methods as appropriate<br>Demands, risks, inconveniences, discomforts of participation in the project<br>Outcomes and benefits of the project<br>Project start, finish, duration
About the investigators / organisation	Researchers conducting the project (including whether student researchers are involved)<br>Organisations which are involved / responsible<br>Organistions which have given approvals<br>Relationship between researchers and particpants and organisations
Participant description	How and why participants are chosen<br>How participants are recruited<br>How many participants are to be recruited
Participant experience	What will happen to the particant, what will they have to do, what will they experience?<br>Benefits to individual, community, and contribution to knowledge<br>Risks to individual, community<br>Consequences of participation
Participant options	Alternatives to participation<br>Whether participation may be for part of project or only for whole of project<br>Whether any of the following will be provided: counselling, post research follow-up, or post research access to services, equipment or goods
Participants rights and responsibilities	That participation is voluntary<br>That participants can withdraw, how to withdraw and what consequences may follow<br>Expectations on participants, consequences of non-compliance with the protocol<br>How to seek more information<br>How to raise a concern or make a complaint
Handling of information	How information will be accessed, collected, used, stored, and to whom data will be disclosed<br>Can participants withdraw their information, how, when<br>Confidentiality of information<br>Ownership of information<br>Subsequent use of information<br>Storage and disposal of information
Unlawful conduct	Whether researcher has any obligations to report unlawful conduct of participant
Financial issues	How the project is funded<br>Declaration of any duality of interests<br>Conspensation entitlements<br>Costs to participants<br>Payments, reimbursements to participants<br>Commercial application of results
Results	What will particpants be told, when and by whom<br>Will individual results be provided<br>What are the consequences of being told or not being told the results of

Core Elements	Issues to consider in participant information
	research<br>How will results be reported / published<br>Ownership of intellectual property and commercial benefits
Cessation	Circumstances under which the participation of an individual might cease<br>Circumstances under which the project might be terminated

Research Specific Elements
Provision of information to participants about the following topics should be considered as may be relevant to the research project.
